# Mortality due to road injuries in the states of India: the Global Burden of Disease Study 1990–2017

**DOI:** 10.1016/S2468-2667(19)30246-4

**Published:** 2019-12-23

**Authors:** India State-Level Disease Burden Initiative Road Injury Collaborators, Rakhi Dandona, G Anil Kumar, Gopalkrishna Gururaj, Spencer James, Joy K Chakma, J S Thakur, Amar Srivastava, Girikumar Kumaresh, Scott D Glenn, Gaurav Gupta, Rinu P Krishnankutty, Rajesh Malhotra, W Cliff Mountjoy-Venning, Parul Mutreja, Anamika Pandey, D K Shukla, Chris M Varghese, Geetika Yadav, K Srinath Reddy, Soumya Swaminathan, Hendrik J Bekedam, Theo Vos, Mohsen Naghavi, Christopher J L Murray, R S Dhaliwal, Lalit Dandona

## Abstract

**Background:**

A systematic understanding of population-level trends in deaths due to road injuries at the subnational level over time for India's 1·4 billion people, by age, sex, and type of road user is not readily available; we aimed to fill this knowledge gap.

**Methods:**

As part of the Global Burden of Diseases, Injuries, and Risk Factors Study, we estimated the rate of deaths due to road injuries in each state of India from 1990 to 2017 based on several verbal autopsy data sources. We calculated the number of deaths and death rate for road injuries by type of road user, and assessed the age and sex distribution of these deaths over time. Based on the trends of the age-standardised death rate from 1990 to 2017, we projected the age-standardised death rate to 2030 to assess if the states of India would meet the Sustainable Development Goal (SDG) target to halve the death rate for road injuries from 2015 by 2020 or 2030. We calculated 95% uncertainty intervals (UIs) for the point estimates.

**Findings:**

In 2017, 218 876 deaths (95% UI 201 734 to 231 141) due to road injuries occurred in India, with an age-standardised death rate for road injuries of 17·2 deaths (15·7 to 18·1) per 100 000 population, which was much higher in males (25·7 deaths [23·5 to 27·4] per 100 000) than in females (8·5 deaths [7·2 to 9·1] per 100 000). The number of deaths due to road injuries in India increased by 58·7% (43·6 to 74·7) from 1990 to 2017, but the age-standardised death rate decreased slightly, by 9·2% (0·6 to 18·3). In 2017, pedestrians accounted for 76 729 (35·1%) of all deaths due to road injuries, motorcyclists accounted for 67 524 (30·9%), motor vehicle occupants accounted for 57 802 (26·4%), and cyclists accounted for 15 324 (7·0%). India had a higher age-standardised death rate for road injury among motorcyclists (4·9 deaths [3·9–5·4] per 100 000 population) and cyclists (1·2 deaths [0·9–1·4] per 100 000 population) than the global average. Road injury was the leading cause of death in males aged 15 to 39 years in India in 2017, and the second leading cause in this age group for both sexes combined. The overall age-standardised death rate for road injuries varied by up to 2·6 times between states in 2017. Wide variations were seen between the states in the percentage change in age-standardised death rate for road injuries from 1990 to 2017, ranging from a reduction of 38·2% (22·3 to 51·7) in Delhi to an increase of 17·0% (0·6 to 34·7) in Odisha. If the trends estimated up to 2017 were to continue, no state in India or India overall would achieve the SDG 2020 target in 2020 or even in 2030.

**Interpretation:**

India's contribution to the global number of deaths due to road injuries is increasing, and the country is unlikely to meet the SDG targets if the trends up to 2017 continue. India needs to implement evidence-based road safety interventions, promote strong policies and traffic law enforcement, have better road and vehicle design, and improve care for road injuries at the state level to meet the SDG goal.

**Funding:**

Bill & Melinda Gates Foundation and Indian Council of Medical Research, Department of Health Research, Ministry of Health and Family Welfare, Government of India.

## Introduction

Road injuries were the eleventh leading cause of death in 2017 globally, with the greatest burden in low-income and middle-income countries.^1^ Halving the number of deaths due to road injuries from 2015 to 2020 is one of the UN Sustainable Development Goals (SDGs).^2^ However, the WHO 2018 global status report on road safety shows very little progress has been made towards this goal.^1^ One of the key recommendations made in the report is to improve the availability of reliable and comprehensive data on the road injury burden to target and monitor progress towards reducing deaths due to road injuries.^1^

The epidemiology of deaths due to road injuries in India has been reported from a nationally representative survey of causes of death in 2001–03 that used verbal autopsy data.^3^ This survey estimated a death rate for road injuries of 26·2 per 100 000 population for males and 5·7 per 100 000 population for females in 2005, and reported wide variations in these rates among the Indian states.^3^ The number of deaths due to road injuries in India was projected to increase to 155 000–183 000 in 2015 on the basis of data from a nationally representative survey of causes of death done in 1998.^4^ Available trends from the administrative source of data on deaths due to road injuries in India, which are known to be under-reported,^5^, ^6^, ^7^, ^8^ also show an increase in the number of deaths due to road injuries.^1^ Road safety in India is under the purview of the Ministry of Road Transport and Highways. Recognising the need to address the large and increasing number of deaths due to road injuries, the Indian Government recently formulated the National Road Safety Policy^9^ and several states have developed corresponding policies.^10^

**Research in context**

**Evidence before this study**

A previous study reported death rates for road injuries in India and its states using verbal autopsy data for 2001–03. Few studies have reported deaths due to road injury from select cities, and some studies have reported death rates for road injuries by state using data from the National Crimes Record Bureau of India, which are known to be an underestimate due to under-reporting. We searched PubMed for published papers on deaths due to road injury in India, Google for the reports in the public domain, and references in these papers and reports, using the search terms “accident, traffic”, “cause of death”, “death”, “epidemiology”, “India”, “mortality”, “road death”, “road injury”, “road traffic injury”, “traffic accident”, and “trends” on May 10, 2019, without language or publication date restrictions. We found a variety of data related to deaths due to road injuries in India and several states in India, but no studies that comprehensively describe population-level estimates of trends in deaths due to road injuries in every state of India over a long period of time, disaggregated by type of road user, age, and sex.

**Added value of this study**

This study provides a comprehensive assessment of the burden of deaths due to road injuries from 1990 to 2017 for each state in India using the same methods over time and between states, describing the trends in death rate for road injuries for the different types of road user, by age and sex. Our findings highlight that the number of deaths due to road injuries in India is increasing, with substantial variations in the magnitude and trends between states and types of road user. We found that deaths rates for road injuries among motorcyclists and cyclists were higher in India than the global average in 2017, and the proportion of deaths due to road injuries among all deaths has increased over time in India. Importantly, we found that road injuries are the leading cause of death among young adult males. Additionally, we found that if the slow decrease in death rate for road injuries continues, no state in India is likely to achieve the SDG 2020 target of halving the death rate for road injuries from 2015 to 2020 even by 2030.

**Implications of all the available evidence**

India will not meet the SDG target of reducing deaths due to road injuries if substantial and rapid improvements in road safety in India are not made. This disaggregated analysis of the trends in deaths due to road injuries in each state of India over the past almost 30 years can provide guidance for national-level and state-level interventions to target populations at increased risk to reduce these deaths.

A comprehensive up-to-date analysis and understanding of the trends in deaths due to road injuries over time in India and its states, and the status of these trends in relation to achieving the SDG target, which is needed to stimulate specific state-level action to reduce deaths due to road injuries, is not available. To address this knowledge gap, we report time trends and variations among the Indian states for deaths due to road injuries from 1990 to 2017, disaggregated by the type of road user, their age and sex, and a comparison of the trend in each state to the SDG target.

## Methods

### Overview

The analysis and findings presented in this Article were produced by the India State-Level Disease Burden Initiative Collaborators as part of Global Burden of Diseases, Injuries, and Risk Factors Study (GBD) 2017.^11^, ^12^, ^13^ The Health Ministry Screening Committee of the Indian Council of Medical Research and the ethics committee of the Public Health Foundation of India have approved the work of this Initiative. A detailed description of the metrics and analytical approaches used in GBD 2017 has been reported elsewhere.^11^, ^12^, ^13^ The methods relevant to this Article are summarised here and described in more detail in the appendix (pp 3–12).

### Cause of death estimation

We used population-representative verbal autopsy data on cause of death from various sources, including the Registrar General of India's Sample Registration System and Survey on Causes of Death, Institute of Health Systems dataset, Indian Council of Medical Research study, and two state-specific verbal autopsy studies covering all age groups (appendix pp 6–8).^3^, ^14^, ^15^ Since 2000, the major data source in India has been the Sample Registration System, which provided data on 455 460 deaths for 2004–13 covering every state in India.^16^ The International Classification of Diseases (ICD) version 10 was used to classify road injuries as pedestrian, cyclist, motorcyclist, motor vehicle occupant and other road injury (appendix p 3).^17^, ^18^ The quality and comparability of the cause of death data were assessed and enhanced through multiple steps in GBD as reported previously.^11^, ^12^, ^16^, ^17^ Several statistical models were used to assess completeness of datasets for calculating all-cause mortality rates. Data were mapped from cause of death sources that had variants of ICD classification to a consistent classification for causes of death. Cause of death codes that could not be mapped based on final classification were redistributed proportionately or using regression models. We formatted data from different sources in a consistent sex and age distribution for standardised analysis. Data on cause of death that were reported in aggregated categories, such as transport or road injuries, were split into subcategories of road user by age-sex groups using patterns from locations with the most similar geography for which this cause of death distribution was available. The Cause of Death Ensemble model (CODEm) was used to assess many potential models that apply different functional forms, such as mixed-effects models and space-time Gaussian Process Regression models, to death rates and cause fractions with varying combinations of predictive covariates. An ensemble of models was selected that performed best on out-of-sample predictive validity tests for each cause of death. A smoothing algorithm was used to avoid erratic estimates. Finally, the CoDCorrect algorithm was used to rescale deaths for each cause to ensure that deaths from all individual causes added up to the number of deaths from all causes generated from the demographic analysis. Because data on cause of death in India were not available for all states for all years of interest, GBD used covariates—variables that have an established association with road injury deaths—to calculate the best possible estimates of the cause of death (appendix pp 9–10).^16^

### Disability-adjusted life-years

The disability-adjusted life-years (DALYs) for road injuries were estimated as the sum of years of life lost (YLLs) and years lived with disability (YLDs).^11^, ^12^, ^13^ YLLs were calculated by multiplying deaths with the remaining life expectancy at the age of death using GBD's standard life table.^11^ YLDs were calculated by multiplying the number of prevalent cases of each health outcome with the disability weight assigned to that health outcome. Because YLLs accounted for most road injury DALYs in India, we focused on deaths due to road injuries in this study.

### Analyses presented in this Article

We present findings for 31 geographical units in India: 29 states, the union territory of Delhi, and union territories other than Delhi. The states of Chhattisgarh, Uttarakhand, and Jharkhand were created from existing larger states in 2000, and the state of Telangana was created in 2014. For trends from 1990 onward, we disaggregated the data for these four new states from their parent states on the basis of data from the districts that now constitute these states. The state of Jammu and Kashmir was divided into two union territories in August, 2019. Because we are reporting findings up to 2017, we report findings for the state of Jammu and Kashmir. We also present findings for three groups of states based on their Socio-demographic Index (SDI) in 2017, as calculated by GBD.^19^ SDI is a composite indicator of development status, which ranges from 0 to 1, and is a geometric mean of the values of the indices of lag-distributed per-capita income, mean education in people aged 15 years or older, and total fertility rate in people younger than 25 years. The three state groups were low SDI (≤0·53), middle SDI (0·54–0·60), and high SDI (>0·60–1·0) states (appendix p 13).^20^

We present trends in the death rate for road injuries from 1990 to 2017 for each Indian state to assess heterogeneity. We estimated the age-standardised death rate by sex for road injury overall, and then specifically for pedestrians, motorcyclists, motor vehicle occupants, cyclists, and other road injuries. We based our age-standardised rates on the GBD global reference population.^11^ We also report crude death rates for road injuries because these data show the actual situation in each state, which is useful for policy makers, whereas age-standardised estimates allow comparisons over time and between states after adjusting for the differences in the age structure of the population. We also report trends in the age-specific death rates between 1990 and 2017 by sex and by type of road user. We provide estimates of the number of deaths due to road injuries by sex for each state in 2017. Estimates are reported with 95% uncertainty intervals (UIs) where relevant, which were based on 1000 draws for each estimate, with the mean regarded as the point estimate and the 2·5th and 97·5th percentiles considered the 95% UI.

We projected the age-standardised death rate for road injuries for India and for each state up to 2030 on the basis of the trends from 1990 to 2017. We calculated the annualised rate of change for the projections from 2018 to 2030 using a weight function that gave a higher weight to the more recent trends. More details on the methods we used for these projections are in the appendix (p 11) and published elsewhere.^21^ We compared the projected age-standardised death rate for road injuries for the years 2020 and 2030 with the SDG target in 2020 for each Indian state (reporting projections for the previously undivided state of Jammu and Kashmir) and then calculated the gap to achieve the SDG 2020 target by 2020 and by 2030.

Kuznets phenomenon, an inverted U-shaped pattern in relation to economic development, has been previously reported for deaths due to road injury globally,^22^ and among the states of India for overall deaths due to road injury.^23^ We assessed the association between the age-standardised death rate for road injury overall and for pedestrians, motorcyclists, motor vehicle occupants, and cyclists in 2017 in each state in India for both sexes combined with their per-capita gross domestic product (GDP)^24^ and with the state-level per-capita vehicles^25^ and we present these results for the best fits.

We compared the number of road injury DALYs and YLLs in India with those of the Organisation for Economic Co-operation and Development (OECD) countries.

### Role of the funding source

Some of the contributors to this Article work with the Indian Council of Medical Research. The other funder, the Bill & Melinda Gates Foundation, of the study had no role in the study design, data collection, data analysis, data interpretation, or writing of the report. The corresponding author had full access to all the data in the study and had final responsibility for the decision to submit for publication.

## Results

India had an increase in the number of deaths due to road injuries of 58·7% (95% UI 43·6–74·7) from 137 901 deaths (127 207–147 874) in 1990 to 218 876 deaths (201 734–231 141) in 2017. This increase was higher than the global increase of 8% (1·3–14·5) from 1·15 million (1·09–1·21) deaths in 1990 to 1·24 million (1·19–1·28) deaths in 2017. The overall age-standardised death rate for road injury in India decreased slightly by 9·2% (0·6–18·3) from 18·9 deaths (17·6–20·2) per 100 000 population in 1990 to 17·2 deaths (15·7–18·1) per 100 000 in 2017, whereas it decreased by 29% globally from 22·3 deaths (21·3–23·4) per 100 000 to 15·8 deaths (15·2–16·3) per 100 000 during this period (Table 1, Table 2). The proportion of deaths due to road injuries among all deaths in India increased from 1·7% (1·5–1·8) in 1990 to 2·2% (2·0–2·3) in 2017, whereas this proportion decreased globally from 2·5% (2·4–2·6) to 2·2% (2·1–2·3). India had 12·0% of the total global deaths due to road injuries in 1990, which increased to 17·6% in 2017 (for full data see the GBD online data visualisation tool).

**Table 1:** Age-standardised death rate for road injuries per 100 000 population in 2017, in India and globally, by type of road user

	**Both sexes**			**Males**			**Females**		
	India	Global	India to global ratio	India	Global	India to global ratio	India	Global	India to global ratio
Overall	17·2 (15·7–18·1)	15·8 (15·2–16·3)	1·09	25·7 (23·5–27·4)	23·9 (22·8–24·6)	1·08	8·5 (7·2–9·1)	8·0 (7·5–8·3)	1·06
Pedestrian	6·4 (5·5–7·1)	6·2 (5·9–6·8)	1·03	8·9 (7·5–10·2)	8·9 (8·2–9·9)	1·00	3·9 (3·3–4·3)	3·6 (3·3–3·9)	1·08
Motorcyclist	4·9 (3·9–5·4)	2·9 (2·5–3·0)	1·69	8·3 (6·4–9·3)	4·9 (4·2–5·2)	1·69	1·5 (1·1–1·8)	0·8 (0·7–0·9)	1·88
Motor vehicle occupant	4·5 (3·9–5·6)	5·8 (5·4–6·0)	0·78	6·2 (5·4–8·2)	8·5 (7·9–9·1)	0·73	2·7 (2·2–3·3)	3·1 (2·8–3·3)	0·87
Cyclist	1·2 (0·9–1·4)	0·9 (0·7–1·0)	1·33	2·1 (1·4–2·5)	1·4 (1·1–1·6)	1·50	0·3 (0·2–0·4)	0·4 (0·3–0·4)	0·75
Other road injuries	0·1 (0·1–0·2)	0·1 (0·1–0·2)	1·00	0·2 (0·1–0·3)	0·2 (0·2–0·3)	1·00	0·1 (0·0–0·1)	0·1 (0·1–0·1)	1·00

Data are age-standardised death rates per 100 000 population, with 95% uncertainty intervals in parentheses.

**Table 2:** Death rates for road injuries in the states of India, 1990–2017

	**1990**		**2017**		**Percentage change in death rate, 1990–2017**	
	Crude death rate	Age-standardised death rate	Crude death rate	Age-standardised death rate	Crude	Age-standardised
**India (1380 million population)**	**15·8 (14·6 to 17·0)**	**18·9 (17·5 to 20·2)**	**15·9 (14·6 to 16·7)**	**17·2 (15·7 to 18·1)**	**0·2% (−9·4 to 10·3)**	**−9·2% (−18·3 to −0·6)**
**Low SDI states (675 million population)**	**16·4 (14·4 to 18·1)**	**19·5 (17·4 to 21·4)**	**15·9 (14·8 to 16·9)**	**18·6 (17·1 to 19·9)**	**−3·3% (−14·0 to 11·3)**	**−4·6% (−15·7 to 8·8)**
Bihar	11·1 (4·9 to 15·3)	13·2 (7·0 to 17·7)	11·4 (10·2 to 12·9)	14·4 (12·8 to 16·1)	2·8% (−26·7 to 135·1)	8·6% (−20·3 to 104·5)
Madhya Pradesh	13·9 (12·0 to 16·2)	16·3 (14·0 to 19·0)	16·1 (14·3 to 18·2)	18·5 (16·3 to 20·9)	15·6% (−5·5 to 38·4)	13·0% (−8·8 to 34·9)
Jharkhand	20·2 (12·8 to 25·7)	25·8 (18·1 to 32·1)	16·9 (15·2 to 18·8)	19·9 (18·0 to 22·0)	−16·5% (−35·5 to 33·0)	−22·9% (−39·0 to 10·7)
Uttar Pradesh	21·5 (18·9 to 24·2)	25·2 (22·1 to 28·4)	18·1 (16·2 to 20·3)	21·6 (19·2 to 24·4)	−15·8% (−29·7 to −0·5)	−14·0% (−28·2 to 1·4)
Rajasthan	16·6 (14·5 to 19·2)	20·3 (17·8 to 23·4)	17·0 (14·8 to 19·3)	19·8 (17·2 to 22·5)	2·3% (−16·2 to 22·3)	−2·1% (−20·5 to 16·6)
Chhattisgarh	14·4 (11·9 to 17·8)	17·9 (14·8 to 22·2)	19·0 (15·6 to 21·7)	21·1 (17·3 to 24·0)	32·4% (−6·0 to 68·4)	18·3% (−17·2 to 50·0)
Odisha	11·1 (9·7 to 13·0)	12·7 (11·0 to 14·9)	14·3 (12·8 to 15·8)	14·8 (13·3 to 16·4)	28·3% (10·0 to 48·4)	17·0% (0·6 to 34·7)
Assam	12·1 (10·5 to 14·0)	14·6 (12·7 to 16·9)	11·9 (10·7 to 13·3)	13·6 (12·2 to 15·2)	−1·6% (−16·5 to 17·0)	−6·6% (−19·6 to 9·2)
**Middle SDI states (387 million population)**	**14·3 (13·1 to 15·5)**	**17·3 (15·9 to 18·7)**	**14·9 (13·4 to 16·0)**	**15·3 (13·7 to 16·5)**	**4·2% (−7·6 to 16·2)**	**−11·4% (−21·9 to −1·4)**
Andhra Pradesh	14·7 (12·0 to 18·0)	17·6 (14·3 to 21·5)	15·9 (12·6 to 19·9)	15·8 (12·4 to 19·8)	7·6% (−16·7 to 40·9)	−10·1% (−31·1 to 17·8)
West Bengal	14·4 (12·1 to 16·7)	18·0 (15·5 to 20·8)	12·2 (10·7 to 13·8)	12·6 (11·1 to 14·2)	−15·3% (−10·3 to 14·7)	−30·2% (−42·6 to −16·4)
Tripura	13·4 (10·8 to 17·1)	16·2 (13·1 to 20·6)	12·8 (10·2 to 16·2)	13·3 (10·8 to 16·6)	−4·2% (−25·6 to 25·7)	−17·8% (−35·7 to 6·6)
Arunachal Pradesh	16·2 (12·7 to 21·1)	21·0 (16·2 to 27·0)	10·6 (8·3 to 14·0)	14·2 (11·2 to 18·1)	−34·4% (−49·5 to −8·7)	−32·2% (−47·6 to −10·2)
Meghalaya	8·6 (6·9 to 10·6)	11·9 (9·6 to 14·5)	7·8 (6·3 to 9·9)	10·0 (8·2 to 12·4)	−9·1% (−28·5 to 19·4)	−15·5% (−33·1 to 8·8)
Karnataka	11·3 (10·0 to 12·9)	13·5 (12·0 to 15·3)	17·0 (13·5 to 19·3)	16·9 (13·6 to 19·2)	49·5% (15·1 to 78·8)	25·2% (−4·4 to 48·5)
Telangana	15·1 (12·2 to 18·8)	18·2 (14·8 to 22·5)	14·1 (11·1 to 17·7)	14·4 (11·4 to 17·9)	−6·6% (−29·4 to 20·8)	−21·3% (−40·6 to 2·0)
Gujarat	13·1 (11·5 to 14·7)	15·5 (13·7 to 17·4)	13·5 (12·1 to 15·0)	14·1 (12·7 to 15·5)	3·1% (−12·4 to 20·9)	−9·6% (−22·6 to 6·0)
Manipur	15·3 (12·7 to 18·2)	20·0 (16·7 to 23·9)	18·4 (14·8 to 23·0)	21·2 (17·0 to 26·0)	20·4% (−4·3 to 53·7)	6·4% (−15·4 to 34·3)
Jammu and Kashmir	26·0 (21·3 to 30·7)	32·3 (26·6 to 38·1)	20·0 (17·2 to 22·8)	21·9 (18·7 to 24·9)	−22·9% (−38·1 to −2·9)	−32·2% (−45·3 to −14·8)
Haryana	17·5 (15·2 to 19·8)	20·9 (18·2 to 23·5)	20·5 (17·9 to 22·8)	21·9 (18·9 to 24·4)	16·6% (−2·3 to 40·7)	4·8% (−12·8 to 25·9)
**High SDI states (318 million population)**	**16·6 (15·4 to 17·9)**	**19·5 (18·1 to 20·9)**	**17·0 (15·1 to 18·3)**	**16·6 (14·7 to 17·9)**	**2·5% (−10·3 to 14·7)**	**−14·8% (−25·3 to −4·6)**
Uttarakhand	26·9 (21·5 to 32·7)	35·7 (28·8 to 43·2)	24·8 (21·2 to 28·4)	26·3 (22·5 to 29·8)	−7·6% (−28·0 to 19·2)	−26·5% (−41·9 to −6·7)
Tamil Nadu	18·2 (16·0 to 20·5)	20·8 (18·4 to 23·4)	20·8 (18·4 to 23·0)	20·0 (17·7 to 22·1)	14·4% (−5·0 to 34·9)	−3·6% (−20·1 to 13·3)
Mizoram	14·3 (11·5 to 17·6)	19·4 (15·7 to 23·8)	14·0 (11·1 to 17·4)	15·9 (12·7 to 19·5)	−2·1% (−27·7 to 28·4)	−17·8% (−39·0 to 6·5)
Maharashtra	16·0 (14·2 to 17·9)	18·9 (16·8 to 21·1)	13·6 (11·9 to 15·1)	13·6 (11·8 to 15·1)	−14·7% (−27·5 to 1·4)	−28·1% (−38·8 to −15·1)
Punjab	20·3 (17·7 to 23·1)	23·3 (20·4 to 26·6)	23·9 (20·8 to 26·9)	22·9 (20·0 to 25·7)	17·6% (−3·7 to 42·4)	−1·7% (−18·9 to 17·8)
Sikkim	11·0 (8·1 to 15·3)	14·9 (11·1 to 20·4)	10·8 (8·8 to 13·4)	11·7 (9·4 to 14·4)	−1·6% (−29·3 to 35·5)	−21·3% (−42·6 to 6·8)
Nagaland	13·9 (11·2 to 17·0)	19·4 (15·5 to 23·5)	11·2 (8·6 to 14·2)	14·1 (10·8 to 17·7)	−19·5% (−37·6 to 5·4)	−27·3% (−44·5 to −5·2)
Himachal Pradesh	14·6 (12·7 to 16·8)	17·3 (15·0 to 19·9)	18·4 (14·3 to 21·4)	17·5 (13·8 to 20·4)	25·5% (−2·4 to 53·8)	1·2% (−20·9 to 23·0)
Union territories other than Delhi	10·7 (9·0 to 12·9)	13·7 (11·5 to 16·5)	13·3 (10·5 to 16·4)	14·0 (11·0 to 17·1)	24·8% (−1·2 to 58·1)	2·3% (−19·5 to 28·8)
Kerala	12·3 (10·7 to 14·1)	14·4 (12·7 to 16·5)	15·9 (13·3 to 18·1)	14·0 (11·7 to 15·8)	30·0% (4·7 to 56·5)	−3·1% (−21·8 to 16·1)
Delhi	14·5 (11·8 to 17·5)	19·0 (15·7 to 22·9)	11·0 (9·1 to 12·7)	11·8 (9·8 to 13·6)	−24·7% (−41·5 to −3·5)	−38·2% (−51·7 to −22·3)
Goa	9·9 (8·2 to 11·8)	11·5 (9·6 to 13·7)	12·3 (9·5 to 15·3)	11·1 (8·7 to 13·8)	24·5% (−7·7 to 62·1)	−3·0% (−28·1 to 26·2)

Data are death rate per 100 000 population, with 95% uncertainty interval in parentheses. States are listed in increasing order of SDI in 2017. SDI=Socio-demographic Index.

In 2017, the overall age-standardised death rate for road injuries varied by up to 2·6 times between states in India, and the crude death rate for road injuries varied by up to 3·2 times (table 2). The age-standardised death rate for road injury was relatively higher in the low SDI state group than in the middle and high SDI state groups in 2017, although the 95% UIs overlapped between the low and high SDI state groups. From 1990 to 2017, the percentage change in age-standardised death rate for road injuries varied widely between the states, from a reduction of 38·2% to an increase of 17·2%. Broadly, the age-standardised death rate for road injury decreased significantly from 1990 to 2017 in the high and middle SDI state groups but not in the low SDI state group during this period (table 2).

Males accounted for 167 830 (76·7%) of deaths due to road injury and females accounted for 51 046 (23·3%). The proportion of deaths due to road injuries among all deaths in males increased in India from 2·3% (2·1–2·5) in 1990 to 3·2% (3·0–3·4) in 2017, and in females increased from 0·8% (0·8–0·9) in 1990 to 1·1% (0·9–1·2) in 2017 (appendix p 14). The age-standardised death rate for road injury by sex in India was similar to the global averages in 2017 (table 1). The age-standardised death rate for road injuries in males was 25·7 deaths (23·5–27·4) per 100 000 population in 2017, varying by up to 2·9 times between states in India (appendix p 15). The highest crude death rates for road injuries in males were in Uttarakhand, Punjab, Tamil Nadu, Jammu and Kashmir, and Himachal Pradesh. The age-standardised death rate for females was 8·5 deaths (7·2–9·1) per 100 000 population in 2017, with no significant change from 1990, varying by up to 3·1 times between states in 2017. The highest crude death rates for road injuries in females in 2017 were in Manipur followed by Jharkhand and Punjab (appendix p 16).

**Figure 1:**
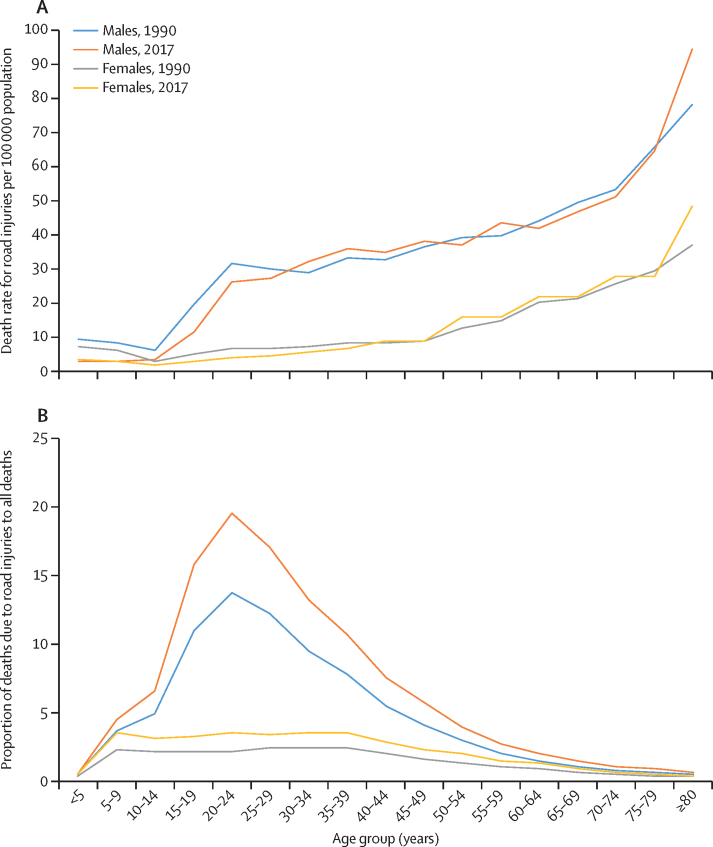
Age-specific death rate for road injuries (A) and the proportion of deaths due to road injuries to total deaths in each age group (B) in India, by sex, for 1990 and 2017

The age-specific death rate for road injuries increased with age for both sexes in 2017 but was higher in males than in females across all age groups (figure 1; appendix p 17). The proportion of deaths due to road injuries among all male deaths increased significantly in the 15–54 years age groups from 1990 to 2017, and for females increased substantially in the 15–39 years age group (appendix p 14). Of the total deaths due to road injuries in 2017, 107 244 (63·9%) of 167 830 in males and 19 614 (38·4%) of 51 046 in females were among those aged 15–49 years (appendix p 18). In the 15–39 years age group in 2017, road injury was the leading cause of death among males and the second leading cause of death in both sexes combined (table 3).

**Table 3:** Proportion of total deaths in India in 2017 due to road injuries in young adults, by sex

	**Proportion of total deaths due to road injuries**	**Rank of road injuries deaths**
**Males**		
15–29 years	17·7% (16·4–19·6)	1
15–39 years	14·4% (13·6–15·2)	1
**Females**		
15–29 years	3·3% (3·0–3·9)	7
15–39 years	3·4% (3·1–3·7)	6
**Both sexes**		
15–29 years	11·1% (10·3–12·2)	2
15–39 years	9·9% (9·4–10·4)	2

Data in parentheses are 95% uncertainty intervals.

**Figure 2:**
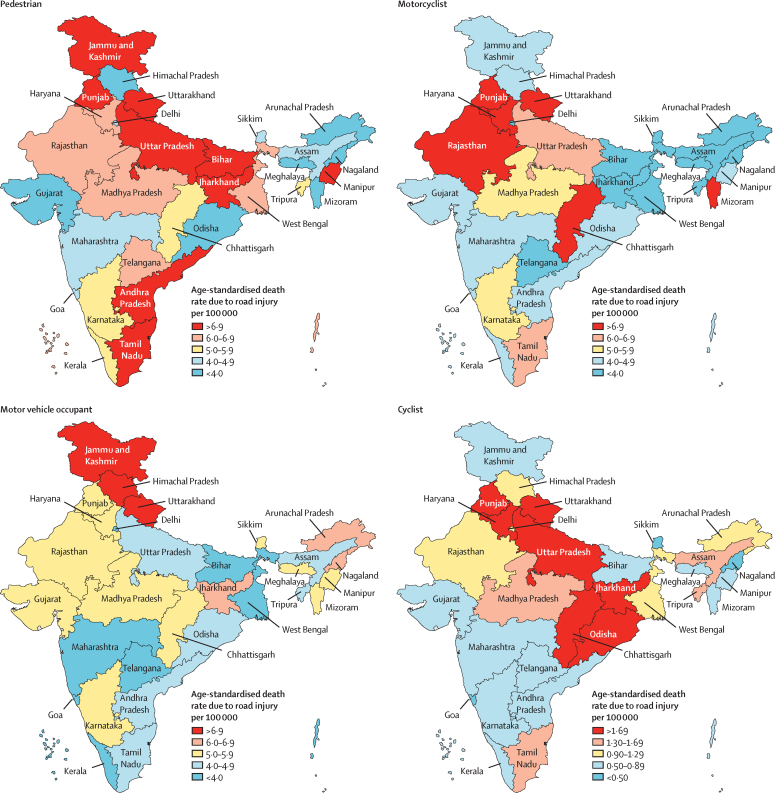
Age-standardised pedestrian, motorcyclist, motor vehicle occupant, and cyclist death rates due to road injuries for both sexes combined in the states of India, 2017

**Figure 3:**
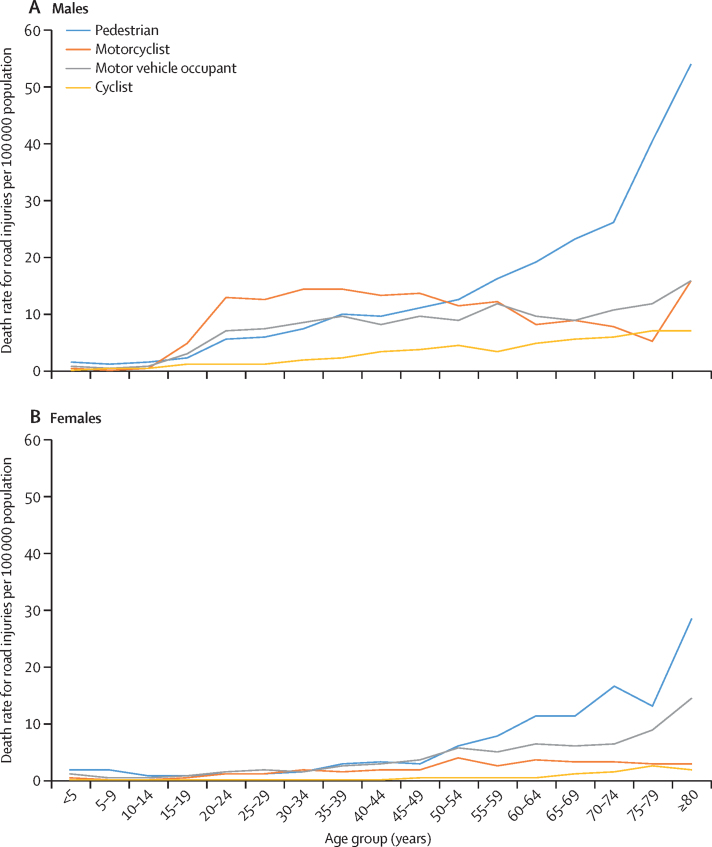
Age-specific death rates for road injuries in India in 2017, by type of road user and by sex

Pedestrian deaths accounted for 76 729 (35·1%) of all deaths due to road injuries in India in 2017, with an age-standardised death rate of 6·4 deaths (5·5–7·1) per 100 000 population, which was similar to the global rate (table 1). Both the crude and age-standardised death rates for road injuries among pedestrians varied by up to four times in 2017 (figure 2; appendix p 15). The highest death rates for road injuries among pedestrians in 2017 were in Manipur, Jammu and Kashmir, Jharkhand, Uttarakhand, Uttar Pradesh, Tamil Nadu, Bihar, Andhra Pradesh, and Punjab, while the lowest rates were in Arunachal Pradesh, Meghlaya, and Mizoram (figure 2; appendix p 15). The variation between the states for this rate was much higher for females (by up to 23 times) than for males (by up to 2·95 times; appendix pp 15–16). Of the total deaths due to road injuries for each sex, the proportion of pedestrian deaths due to road injuries in females (22 885 [44·8%] of 51 046) was higher than in males (53 845 [32·1%] of 167 830; appendix p 19). The age-specific death rate for road injuries among pedestrians increased with age in both sexes in 2017 compared with 1990, particularly in males older than 20 years and in females older than 50 years (figure 3; appendix pp 20–21).

Deaths among motorcyclists accounted for 67 524 (30·9%) of all deaths due to road injuries in India in 2017, with an age-standardised death rate of 4·9 deaths (3·9–5·4) per 100 000 population, which was 69% higher than the global average (table 1). Between states in India in 2017, the age-standardised death rate for road injuries among motorcyclists varied by up to 7·3 times, and crude death rate varied by up to 8·4 times (appendix p 15). This age-standardised rate was the highest in Punjab, Uttarakhand, Haryana, Chhattisgarh, Rajasthan, and Mizoram (figure 2; appendix p 15). The crude death rate for road injuries among motorcyclists in India in 2017 was 5·9 times higher in males than in females (appendix pp 15–16). Of the total deaths due to road injuries in each sex, the proportion of motorcyclist deaths due to road injuries among males (58 016 [34·6%]) was higher than that among females (9508 [18·6%]; appendix p 19). In males, the death rate for road injuries among motorcyclists increased sharply after age 15 years, remained high at ages 20–59 years, and then decreased slightly between ages 60 and 75 years, whereas the rate in females was higher after age 30 years than in the younger age groups (figure 3; appendix p 21).

Deaths among motor vehicle occupants accounted for 57 802 (26·4%) of all deaths due to road injuries in India in 2017, with an age-standardised death rate of 4·5 deaths (3·9–5·6) per 100 000 population, which was 28% lower than the global average (table 1). The age-standardised death rate for road injuries among motor vehicle occupants varied by up to 3·6 times between states in India in 2017, and the crude death rate varied by up to 3·4 times (appendix p 15). This age-standardised rate was the highest in Himachal Pradesh, Uttarakhand, and Jammu and Kashmir (figure 2; appendix p 15). The crude death rate for road injuries among motor vehicle occupants in India in 2017 was 2·4 times higher in males than in females (appendix pp 15–16). Of the total deaths due to road injuries in each sex, the proportion of motor vehicle occupant deaths due to road injuries was lower among males (41 401 [24·7%]) than that among females (16 402 [32·1%]; appendix p 19). The age-specific death rate for road injuries among motor vehicle occupants in males increased after age 20 years, with slight variations in adulthood and a relatively high rate in the oldest age groups, whereas in females the death rate steadily increased after age 20 years (figure 3; appendix p 21).

Deaths among cyclists accounted for 15 324 (7·0%) of all road injury deaths in India in 2017, with an age-standardised death rate of 1·2 deaths (0·9–1·4) per 100 000 population, which was 33% higher than the global average (table 1). The age-standardised death rate for road injuries among cyclists varied by up to 5·7 times between states in India in 2017, and the crude death rate varied by up to 7 times. This age-standardised rate was the highest in Punjab, Uttarakhand, Uttar Pradesh, Chhattisgarh, Odisha, Haryana, and Jharkhand (figure 3; appendix p 15). The crude death rate for road injuries among cyclists in India in 2017 was 6·3 times higher in males than in females (appendix pp 15–16). The proportion of deaths due to road injuries among cyclists in males (13 497 497 497 [8·0%]) was higher than that in females (1827 [3·6%]), with variation across the states (appendix p 19). The age-specific death rate for road injuries among cyclists in males slightly increased after age 30 years whereas the rate in females was generally low in most age groups (figure 3; appendix p 21).

Death rates for other road injuries by sex are in the appendix (p 19).

Of all road users, a significant but weak inverted U-shaped relationship was only seen for the age-standardised death rate for road injuries among motor vehicle occupants for both sexes combined with the state per-capita GDP (R^2^=0·20; p=0·020; appendix pp 22–24) and for the state per-capita vehicles (R^2^=0·20; p=0·044; appendix pp 25–27).

**Figure 4:**
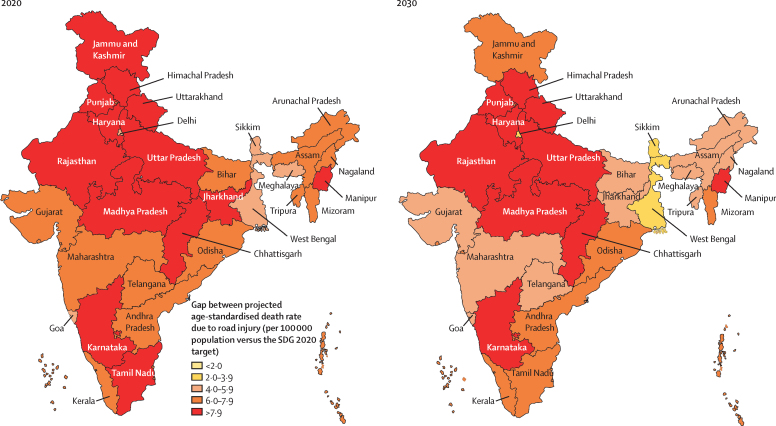
Gap between the projected age-standardised death rate for road injuries versus the SDG target for the states of India, 2020 and 2030 SDG target is to halve the death rate for road injuries from 2015 to 2020. The projections for 2020 and 2030 are reported for the previous undivided state of Jammu and Kashmir. SDG=Sustainable Development Goal.

If the trends estimated up to 2017 were to continue, the projected age-standardised death rate for road injuries for India in 2020 would be 16·8 (15·0–17·9) per 100 000 population, which is substantially higher than the SDG target of 8·9 per 100 000 in 2020 (appendix p 28). Furthermore, none of the states in India would meet their respective SDG 2020 targets (figure 4; appendix p 28). Even if the SDG 2020 target was considered as the target for 2030, if the trends estimated up to 2017 continue, none of the states in India would meet the target in 2030, although the gap would be somewhat smaller than in 2020 (figure 4; appendix p 28).

Of the total estimated DALYs for road injuries in India, the proportion due to YLLs decreased slightly from 93·7% in 1990 to 89·2% in 2017. By contrast, the proportion of total estimated DALYs for road injuries in the OECD countries due to YLLs was 82·8% in 1990 and 69·5% in 2017 (for full data see the GBD online data visualisation tool).

## Discussion

The global death rate for road injuries has decreased substantially from 1990 to 2017, but only slightly in India, with India's share of the number of global deaths due to road injury increasing during this period. Motorcyclists and cyclists have a higher death rate for road injuries in India than the global average. The contribution of deaths due to road injuries to the total number of deaths in India has increased, and road injuries are the leading cause of death among young adult males in India. The death rates for road injuries vary substantially between the states of India. The trends presented here for each state in India over time for the different road users, age groups, and sexes can inform prevention policies for road injuries and monitoring of the road injury burden at the state level. Importantly, we found that, if the trends up to 2017 were to continue, India and its states are unlikely to achieve the SDG target of reducing the death rate for road injuries by half from 2015 to 2020, or even up to 2030.

Pedestrians, motorcyclists, and cyclists are known to be vulnerable road users because they are less protected than the driver or passengers of a car. The higher death rate for road injuries in India among motorcyclists and cyclists than the global average reflects a higher proportion of these types of road users in India and indicates that the road infrastructure and vehicle design in India gives priority to people with cars and not to those who might not be able to afford a car, so affecting their safety.^26^, ^27^, ^28^, ^29^ This pattern of ignoring the vulnerable road users in the planning, designing, and operation of roads and in vehicle design is prevalent in India, and is also seen in many low-income and middle-income countries.^1^ The poor enforcement of speed limits, drink-driving laws, motorcycle helmet use, and seatbelt laws contribute further to the burden of deaths due to road injuries in India.^1^, ^30^ The Government of India has attempted to address these issues through the recently amended Motor Vehicle Act Amendment Bill of 2019.^31^ This Bill has increased fines by up to five times and now includes imprisonment as a deterrent against traffic violations, recall of defective vehicle parts by automobile companies, holding builders accountable for poor quality of road infrastructure, and making vehicle owners criminally liable for violations committed by juvenile drivers. Because the Bill came into effect in September, 2019, we cannot yet comment on its possible impact, but, notably, the Bill has not been implemented uniformly by all the states.^32^, ^33^, ^34^

In 2017, males accounted for 77% of the deaths due to road injuries, and they had a three-times higher age-standardised death rate than females in India. A higher road injury mortality in males than in females has been reported in India previously,^3^, ^6^, ^35^, ^36^, ^37^ and has also been reported globally across all age groups because females have a lower risk of road injury due to cultural or economic reasons and lower risk taking behaviour than males do.^38^ With deaths due to road injuries being the leading cause of death in males aged 15–39 years and the second leading cause of death in this age group for both sexes combined in 2017, road injuries can have far-reaching economic implications including loss of the primary breadwinner, funeral costs, and costs of care, which can push families into poverty.^38^, ^39^, ^40^, ^41^, ^42^ A substantial proportion of these deaths due to road injuries are likely to occur during commuting to and from work, as is shown in developed countries,^43^ but little information on commuting patterns are available from India.^44^, ^45^, ^46^ Age-specific death rates for road injury increased with older age. Ageing is one of the crucial risk factors that affects road injury outcomes,^47^, ^48^, ^49^ but specific studies on the road use patterns of older people or their injury outcomes are not readily available from India. Urgent further work is required to understand patterns of road use by age and type of road user to facilitate effective policy making.

Rapid urbanisation and economic growth in India have led to substantial increases in vehicle density but also heterogeneity in roads users. The required infrastructure and levels of traffic law enforcement are lagging behind.^7^, ^50^ In this study, we explored the Kuznets phenomenon for overall death rate for road injuries and by type of road users separately with state per-capita GDP and per-capita vehicles, which highlighted associations that are hidden when only overall death rate for road injuries is considered. An inverted U-shaped relationship with the state per-capita GDP and per-capita vehicles was seen only for the age-standardised death rate for motor vehicle occupants. An inverted U-shaped relationship has been reported previously with state-level net domestic product for overall death rate for road injuries using administrative data.^23^ Our findings, on the one hand, highlight the need to understand this relationship better, and on the other hand, indicate the urgent need for India and its states to improve investments in road safety in line with rising GDP to address the increasing number of deaths due to road injury.^1^, ^7^ With the per-capita GDP being an important determinant of per-capita vehicles,^22^, ^23^, ^51^ the government need to invest in public transport systems that reduce individual motorcycle and car usage in India.^52^ Towards this aim, some Indian cities are at varying stages of implementing the Bus Rapid Transport Projects,^53^ but barriers to successful implementation of such projects need to be addressed effectively, including gaining acceptance from the community, in particular from those who do not use public transport.^54^, ^55^ Furthermore, global policies that support a modal shift away from private motor vehicles towards walking, cycling, and low-emission public transport can positively influence the overall health of city populations in addition to reducing road injuries across the world, as well as in India.^52^

The YLLs accounted for an overwhelming 89·2% of the road injury DALYs in India in 2017, whereas this proportion was 69·5% in OECD countries because of better trauma care, highlighting the need to address the poor access to and inadequate quality of trauma care, including pre-hospital care in India.^28^, ^56^, ^57^, ^58^, ^59^ To respond to these challenges, some recent developments are worth mentioning. India enacted the Good Samaritan Law in 2016 to increase involvement of bystanders to help those involved in road traffic accidents;^60^ however, awareness and implementation of this law 2 years after its enactment is low.^60^ In 2014, the government also initiated a scheme of cashless access to provide care within the first hour of a road injury. The Government of India has initiated steps towards improving the quality of data and trauma care through the National Injury Surveillance, Trauma Registry and Capacity-Building Centre under the Ministry of Health and Family Welfare.^9^

Concerted efforts are needed at the state level to address these issues to reduce the number of deaths due to road injuries.^10^, ^28^ With each state developing their respective road safety policies, the detailed fatality data for road injuries by sex, age, and type of road user presented in this Article could facilitate appropriate targeting of population subgroups, planning for relevant interventions on the basis of the pattern of the mortality burden for road injuries in a given state, and facilitate setting of relevant targets.^10^

The administrative source of data on deaths due to road injuries in India is police records, which are documented in reports by the Ministry of Road Transport and Highways and the National Crimes Record Bureau.^61^ Despite the same source of data, varying numbers of deaths are reported in the two reports.^61^, ^62^ Both these reports provide information on specific risk factors, spatial distribution, and time of occurrence of traffic collisions, but in heavily tabulated fixed formats that restrict the understanding of these data and their use in further analyses.^63^ Furthermore, under-reporting of deaths due to road injuries in police records and the possible reasons for under-reporting have been documented, particularly for vulnerable road users.^5^, ^8^, ^27^, ^61^, ^64^ An integrated road accident database project was launched in early 2019 by the Government of India to address these under-reporting issues; however, few details are currently available.^65^ In addition to the substantial effort needed to improve the quality of information collected on road injuries by the police through standardised formats and training,^66^ meaningful insights for action can also be generated if the anonymised individual-level data from police records can be made available in the public domain for analysis.^67^

The general limitations of GBD methods and those for estimating injuries, including road injuries, have been published elsewhere.^11^, ^12^, ^13^, ^16^ A specific limitation for estimating deaths due to road injuries in India is an incomplete Medical Certified Cause of Death system that covers only 22% of all deaths in India, covering mainly urban areas, and with variable coverage across the states.^68^ Verbal autopsy data on cause of death are generally considered a reasonable alternative for cause of death distribution at the population level.^16^, ^69^, ^70^, ^71^ The findings in this Article are based on several sources of verbal autopsy data since the 1980s. The Registrar General of India's Survey of Causes of Death has collected rural verbal autopsy data on cause of death since 1982, superseding the earlier Model Registration Scheme.^15^ This survey was merged with the Registrar General of India's Sample Registration System verbal autopsy cause of death system since 1999, covering both rural and urban areas. The cause of death assignment due to road injuries has been reported to be of reasonable quality in both the Survey of Causes of Death and Sample Registration System.^3^, ^72^ Nevertheless, improvement in the coverage and quality of the Medical Certified Cause of Death system is essential in India for a more robust understanding of causes of death. Because data on deaths due to road injuries for all states were not available for the entire duration covered in this study, we used the association with covariates to generate estimates for all states for all years. Under-reporting of deaths due to road injuries is known and misclassification is possible.^5^, ^8^, ^64^ The GBD methods address this undercounting in deaths by redistribution of improbable causes of deaths to the likely underlying causes, which is a substantial strength of our study. The substantial contribution of a network of experts from India in the analysis and interpretation of the findings is another strength of this study.

In conclusion, increasing motorisation is a major challenge faced by India as the country's income and urban population continue to grow. The small reduction in the death rate for road injury in India between 1990 and 2017 is a concern. India is not likely to meet the SDG 2020 target to reduce deaths due to road injuries even by 2030 if the trends observed continue. Much is known about which interventions work, such as the need for strong policies and traffic law enforcement, better road and vehicle designs, and multisectoral approaches to address the road injury burden.^1^, ^28^, ^38^, ^73^ India needs to promote these approaches and scale-up progress in evidence-based interventions to improve road safety, enhance the involvement of the health system to deal with road injuries, and improve availability of quality actionable data. The findings in this Article present an opportunity for the national and state governments and other stakeholders in India to plan improved targeted interventions to achieve the SDG target by 2030.

**This online publication has been corrected. The corrected version first appeared at thelancet.com/public-health on February 4, 2020**
