# Correction to *Lancet Public Health* 2020; 5: e86–98

**DOI:** 10.1016/S2468-2667(20)30012-8

**Published:** 2020-02-04

*India State-Level Disease Burden Initiative Road Injury Collaborators. Mortality due to road injuries in the states of India: the Global Burden of Disease Study 1990–2017*. Lancet Public Health *2020; **5:** e86–98—*In this Article, Geetika Yadav should have been included in the list of India State-Level Disease Burden Initiative Road Injury Collaborators. Her name has been added to the list and the Affiliations and Declaration of interests sections have been updated. Additionally, in figure 2, the key was omitted from panels B, C and D. These corrections have been made as of Feb 4, 2020.

