# Supplementary material for: Mortality due to road injuries in the states of India: the Global Burden of Disease Study 1990–2017
Source: Lancet Public Health. 2019 Dec 23;5(2):e86–98. doi: 10.1016/S2468-2667(19)30246-4 (PMC7098470; doi:10.1016/S2468-2667(19)30246-4)
Supplement: Supplementary appendix [file mmc1.pdf]

THE LANCET
Public Health

Supplementary appendix

This appendix formed part of the original submission and has been peer reviewed. We post it as supplied by the authors.

Supplement to: India State-Level Disease Burden Initiative Road Injury Collaborators. Mortality due to road injuries in the states of India: the Global Burden of Disease Study 1990–2017. *Lancet Public Health* 2019; published online Dec 23. https://doi.org/10.1016/S2468-2667(19)30246-4.

Deaths due to road injuries in the states of India:

the Global Burden of Disease Study 1990-2017

India State-Level Disease Burden Initiative Road Injury Collaborators

Web Appendix

Correspondence to: Prof. Rakhi Dandona, rakhi.dandona@phfi.org

Table of contents

1. GBD 2017 road injuries mortality estimation methods 3
2. Grouping of the states of India based on SDI, 2017 13
3. Proportion of road injury deaths among the total deaths in each age group in males and females in India, 1990 and 2017 14
4. Road injury death rate by type of road users in the states of India, 2017 15
5. Age-specific road injury death rate among males and females in India, 1990 and 2017 17
6. Proportion of total road injury deaths in each age group among males and females in India, 1990 and 2017 18
7. Road injury deaths and their proportion by type of road users among males and females in the states of India, 2017 19
8. Age-specific road injury death rate by type of road users in India, 2017 20
9. Relationship between the age-standardised death rate for overall, motor vehicle occupant, motorcyclist, pedestrian, and cyclist road injury with the per capita GDP in the states of India, 2017 22
10. Relationship between the age-standardised death rate for overall, motor vehicle occupant, motorcyclist, pedestrian and cyclist road injury with the per capita vehicles in the states of India, 2017 25
11. Gap between the projected road injury ASDR versus the SDG target for the states of India in 2020 and 2030 28

1. GBD 2017 road injuries mortality estimation methods

The material presented here is adapted from the following sources:

- GBD 2017 Causes of Death Collaborators. Global, regional, and national age-sex-specific mortality for 282 causes of death in 195 countries and territories, 1980–2017: a systematic analysis for the Global Burden of Disease Study 2017. *Lancet* 2018; 392: 1736–88.
- Global, regional, and national age-sex-specific mortality and life expectancy, 1950–2017: a systematic analysis for the Global Burden of Disease Study 2017. *Lancet* 2018; 392: 1684-735.

The GBD cause list is organised hierarchically into four levels. At each level of the hierarchy, the set of causes is mutually exclusive and collectively exhaustive. The broad group "Injuries" is at level 1. Level 2 includes three sub-types of injuries that are transport injuries, unintentional injuries, and self-harm and interpersonal violence. Level 3 under "transport injuries" includes road injuries and other transport injuries. Level 4 under "road injuries" includes pedestrian road injuries, cyclist road injuries, motorcyclist road injuries, motor vehicle road injuries, and other road injuries.

A. List of ICD codes mapped to the GBD road injuries mortality list

The codes used by GBD Study 2017 from the 9th and 10th revisions of the International Statistical Classification of Diseases and related health problems (ICD) for road injuries mortality are listed below:

Causes	ICD 10	ICD 9
Road injuries	V01 to V04.99<br>V06 to V80.929<br>V82 to V82.9<br>V87 - V87.1*<br>V87.2 -V87.3<br>V87.4 to 87.9*<br>V88 - V88.1*<br>V88.4 to V88.9*<br>V89 to V89.9*	E810.0-E810.6, E811.0-E811.7, E812.0-E812.7, E813.0-E813.7, E814.0-E814.7, E815.0-E815.7, E816.0-E816.7, E817.0-E817.7, E818.0-E818.7, E819.0-E819.7, E820.0-E820.6, E821.0-E821.6, E822.0-E822.7, E823.0-E823.7, E824.0-E824.7, E825.0-E825.7, E826.0-E826.1, E826.3-E826.4, E827.0, E827.3-E827.4, E828.0, E828.4, E829.0-E829.4
Pedestrian road injuries	V01 to V04.99<br>V06 to V09.9	E811.7, E812.7, E813.7, E814.7, E815.7, E816.7, E817.7, E818.7, E819.7, E822.7, E823.7, E824.7, E825.7, E826.0, E827.0, E828.0, E829.0
Cyclist road injuries	V10 to V19.9	E800.3, E801.3, E802.3, E803.3, E804.3, E805.3, E806.3, E807.3, E810.6, E811.6, E812.6, E813.6, E814.6, E815.6, E816.6, E817.6, E818.6, E819.6, E820.6, E821.6, E822.6, E823.6, E824.6, E825.6, E826.1
Motorcyclist road injuries	V20 to V29.9	E812.3, E813.2-E813.3, E814.2-E814.3, E815.2-E815.3, E816.2-E816.3, E817.2-E817.3, E818.2-E818.3, E819.2-E819.3, E820.2-E820.3, E821.2-E821.3, E822.2-E822.3, E823.2-E823.3, E824.2-E824.3, E825.2-E825.3
Motor vehicle occupant road injuries	V30 to V79.9<br>V87.2 - V87.3	E810.0-E810.1, E811.0-E811.1, E812.0-E812.1, E813.0-E813.1, E814.0-E814.1, E815.0-E815.1, E816.0-E816.1, E817.0-E817.1, E818.0-E818.1, E819.0-E819.1, E820.0-E820.1, E821.0-E821.1, E822.0-E822.1, E823.0-E823.1, E824.0-E824.1, E825.0-E825.1
Other road injuries	V80 toV80.929<br>V82 to V82.9	E810.4-E810.5, E811.4-E811.5, E812.4-E812.5, E813.4-E813.5, E814.4-E814.5, E815.4-E815.5, E816.4-E816.5, E817.4-E817.5, E818.4-E818.5, E819.4-E819.5, E820.4-E820.5, E821.4-E821.5, E822.4-E822.5, E823.4-E823.5, E824.4-E824.5, E825.4-E825.5, E826.3-E826.4, E827.3-E827.4, E828.4, E829.4

*These codes were treated as garbage codes and redistributed based on regression as detailed in appendix p 8

GBD data and analysis framework

The overview of data inputs and analysis framework for GBD is shown in the following flowchart:

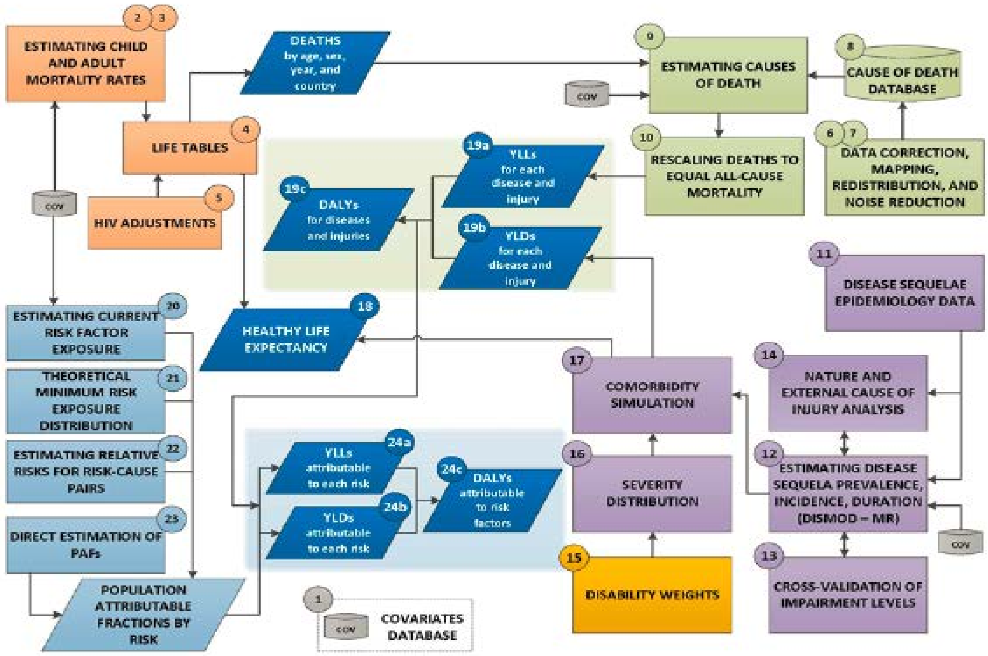

YLLs is years of life lost. YLDs is years lived with disability. DALYs is disability-adjusted life-years. PAFs is population attributable fractions.
Rectangular boxes represent analytical steps, cylinders represent databases, and parallelograms represent intermediate and final results.

The flowchart above illustrates the flow of the key components of the GBD estimation process, including:

1. Incorporation of appropriate covariates (step 1).
2. All-cause mortality estimation (steps 2-5): the data come from sources such as censuses, surveys and vital registrations. The all-cause mortality estimation process (steps 2-4) can be divided into four distinct but interconnected areas: child mortality and adult mortality between ages 15 and 60, estimation of a complete set of age-specific death rates, estimation of HIV mortality and final estimates of age-specific mortality including HIV and fatal discontinuities (also known as mortality shocks) (step 5).
3. Causes of death estimation (steps 6-9): cause of death data are derived from vital registrations, verbal autopsy studies, mortality surveillance and, for selected causes, police records, crime reports and data collection systems for deaths due to conflict and natural disasters (step 7). Extensive data corrections and redistributions of ill-defined causes are made to correct for measurement bias between data sources. Cause of death ensemble modelling (CODEm), an ensemble model, is a systematized approach to analysing cause of death data for all but a few causes (step 9). CODEm explores a wide range of modelling approaches and varying predictive covariates to find an ensemble of best-performing models based on statistical tests. To do so, 30% of the data are withheld from each model and the model fit is evaluated by how well it covers the data that were left out. By repeating this process many times over the best performing models are selected. As all results in GBD are estimated 1,000 times over to propagate all sources of uncertainty, among the 1,000 runs we end up with an ensemble of up to 100 or more different types of models and covariates that are selected.
4. Rescaling deaths to equal all-cause mortality (step 10): as all these estimates are made separately for each disease and injury, the sum of these could exceed or fall below the all-cause mortality estimated from the demographic analyses of steps 2 to 5. Therefore, all deaths by age, sex, geography, year and cause to match the all-cause death estimates (this process is called CoD Correct).
5. Estimation of disease sequelae prevalence, incidence, and duration (steps 11-12): population surveys, cohort studies, administrative records of hospitalisations and other health service encounters, disease registries, notifications, surveillance systems are the main data sources for non-fatal estimation (step 11). Extensive corrections of data to deal with measurement bias arising from study design or case definitions are applied. DisMod-MR 2.1 is the main analytical tool for non-fatal estimation (step 12). It is a Bayesian meta-regression software program that uses a lognormal model. The meta-regression component allows

corrections for known sources of measurement error. Its core function is to make estimates of prevalence and incidence of disease that are consistent with data on mortality risk and remission (defined in GBD as the ‘cure rate’). For a select number of causes that do not fit well in the three state model (alive without disease, prevalent case of disease and death) of DisMod-MR 2.1, was used as alternative modelling strategies.

6. Cross-validation of impairment levels (step 13): for a number of impairments in GBD terminology, such as anaemia, heart failure, hearing and vision loss, we first estimate the total levels of prevalence and incidence and then ensured that all sequelae of diseases that lead to this impairment add up to the total.
7. Analysis of the nature and external cause of injury is done separately (step 14). Assignment of severity distributions for the main disabling conditions (step 15): in GBD terminology sequelae are the disabling consequences for which we make estimates. All sequelae are defined to be mutually exclusive and collectively exhaustive. Many diseases have sequelae with a gradation by severity such as mild, moderate and severe dementia. Often the epidemiological data on severity distribution is sparse. Therefore, at first model the epidemiology of all cases of disease and then apply a severity distribution from the sparser data.
8. Assignment of disability weights for health states (step 16): each sequela is matched with a health state or combination of health states for which we have a disability quantifies the relative severity.
9. Disability weights were derived from population and internet surveys of over 60,000 respondents answering pair-wise comparison question of random combinations of health states. Each pair of health states was described with brief lay descriptions highlighting the main symptoms and impairments. Respondents were asked to nominate the ‘healthier’ of each presented pair. Analytical methods exist to formalise the intuition that if the majority of respondents nominate one health state in a pair as the healthier these lie farther apart on a severity scale than pairs assigned similar proportions as the healthier. In order to anchor estimates on a 0-1 scale of severity, a subset of respondents was asked additional population health equivalence questions on a selection of health states. These questions ask for a choice of the greater amount of health produce by two health programs; one that prevented sudden death in 1,000 persons and another that prevented the onset of a GBD health state for the rest of 2,000, 5,000 or 10,000 persons’ lives.
10. Simulation of comorbidity (step 17): the last step of non-fatal estimation is a microsimulation (‘COMO’) to deal with comorbidity. For every age, sex, geography and year, 40,000 hypothetical persons are generated who have none, one or more of the GBD sequelae. In those with multiple sequelae their combined level of disability is estimated multiplicatively. That means we assume the disability from having two health states is less than the sum of the corresponding disability weights. This avoids assigning disability greater than one to any individual which would indicate that person is worse off than being dead.
11. Estimation of healthy life expectancy (step 18): health life expectancy is estimated from the life tables generated in step 4 and the all-cause YLD rates from step 19b.
12. Computation of YLLs, YLDs, and DALYs from diseases and injuries with uncertainty (steps 19a-19c): YLLs (step 19a) are estimated as the product of counts of death by ages, sex, geography, year and cause and a normative life expectancy at the age of the death. The GBD standard life expectancy used as this norm is a compilation of the lowest observed mortality rates by age in all mortality data collections of populations greater than 5 million. The standard life table reflects a life expectancy at birth of 86.6 years. YLDs are the output from COMO (step 19b). DALYs are the simple addition of YLLs and YLDs (step 19c).
13. Risk factor estimation (steps 20-24): GBD 2017 also makes estimates for individual and combined risk factors. This involves estimation of risk factor exposure (step 20); the formulation of a minimum level of exposure to each risk that is associated with the least amount of health loss (step 21); derivation of relative risks of disease outcomes for each pair of a risk factor and a disease or injury for which there is judged to be sufficient evidence of a causal relationship (step 22); and the estimation of population attributable fractions of disease caused by each risk factor. For a few risk-outcome pairs it is hard to define exposure and a corresponding risk while directly observed proportions of disease are available, such as for the proportion of HIV/AIDS due to unsafe sex or injecting drug use (step 23). For combinations of risks how much of the risk is mediated through other risks (step 24) was assessed. For instance, all of the effect of high salt intake is mediated through elevated blood pressure and part of the risk of increased body mass index is through elevated blood pressure, cholesterol or fasting plasma glucose.
14. Computation of YLLs, YLDs, and DALYs attributable to risk factors (steps 25a-25c): YLLs, YLDs and DALYs attributable to each risk factor are generated by multiplying population attributable fractions with disease estimates (steps 25a-c).

B. Modelling process

The standardised methods and data of the Global Burden of Disease Study (GBD 2017) facilitate a comparative assessment of patterns and trends in road injuries mortality. The data and methods in GBD 2017 represent substantive improvements to previous iterations of the GBD study including additional data sources, advances in modelling strategies, and the refinement of a continuous scale the Socio-demographic Index (SDI) to measure health trends relative to socio-economic factors between locations.

The GBD 2017 study includes estimates of mortality due to 359 causes of death, and 84 risk factors by age, sex, and location between 1990 and 2017 for 195 countries and territories. A detailed description of the data sources and processing steps for the cause of death database can be found in the appendix to the GBD 2017 paper "Global, regional, and national age-sex specific mortality for 282 causes of death in 195 countries and territories, 1980–2017: a systematic analysis for the Global Burden of Disease Study 2017".[1] Here, we provide an overview of the methods employed by the GBD Study with details specific to the estimation of fatal outcomes of road injuries.

Road injuries mortality data sources in India

The data sources used for estimation of road injury deaths in India are listed below.

Data years	Data sources	States
2004-2013	Office of the Registrar General & Census Commissioner, Ministry of Home Affairs, Government of India. India SRS Verbal Autopsy 2004-2013. New Delhi, India: Office of the Registrar General and Census Commissioner. [Data shared for this analysis]	Andhra Pradesh, Arunachal Pradesh, Assam, Bihar, Chhattisgarh, Delhi, Goa, Gujarat, Haryana, Himachal Pradesh, Jammu and Kashmir, Jharkhand, Karnataka, Kerala, Madhya Pradesh, Maharashtra, Manipur, Meghalaya, Mizoram, Nagaland, Odisha, Punjab, Rajasthan, Sikkim, Tamil Nadu, Tripura, Union Territories other than Delhi, Uttar Pradesh, Uttarakhand, and West Bengal
1980-1995	Office of the Registrar General & Census Commissioner, Ministry of Home Affairs, Government of India. India Survey of Causes of Death 1980-1995. New Delhi, India: Office of the Registrar General and Census Commissioner.	Andhra Pradesh, Assam, Bihar, Delhi, Goa, Gujarat, Haryana, Himachal Pradesh, Karnataka, Kerala, Madhya Pradesh, Maharashtra, Manipur, Odisha, Punjab, Rajasthan, Tamil Nadu, Union Territories other than Delhi, and Uttar Pradesh
1980-1998	Institute of Health Systems. India Cause of Death Dataset Version 1.3 1980-1998. Hyderabad, India: Institute of Health Systems; 2002.	Andhra Pradesh, Assam, Bihar, Delhi, Goa, Gujarat, Haryana, Himachal Pradesh, Karnataka, Kerala, Madhya Pradesh, Maharashtra, Manipur, Odisha, Punjab, Rajasthan, Tamil Nadu, Union Territories other than Delhi, and Uttar Pradesh
2003	Indian Council of Medical Research (ICMR). India Study on Causes of Death by Verbal Autopsy 2003. New Delhi, India: ICMR. [Data shared for this analysis]	Assam, Bihar, Maharashtra, Rajasthan, and Tamil Nadu
1992	INDEPTH Network. Africa, Asia, Oceania - INDEPTH Network Cause-Specific Mortality - Release 2014. Accra, Ghana: INDEPTH Network; 2014.	Haryana and Maharashtra
2003-2004	Joshi R, Cardona M, Iyengar S, Sukumar A, Raju CR, Raju KR, Raju K, Reddy KS, Lopez A, Neal B. Chronic diseases now a leading cause of death in rural India – mortality data from the Andhra Pradesh Rural Health Initiative. Int J Epidemiol. 2006; 35: 1522-9.	Andhra Pradesh
2011-2014	Public Health Foundation of India. Cause of Death Estimation Study in Bihar 2011-2014. New Delhi, India: Public Health Foundation of India. [Data shared for this analysis]	Bihar

The Sample Registration System (SRS) in India is operated by the Office of the Registrar General of India working under the Ministry of Home Affairs, Government of India.[2] Cause of death data from SRS verbal autopsy on 455,460 deaths from the rural and urban populations of every state of India from 2004 to 2013 were provided by the Office of the Registrar General of India for analysis by the India State-Level Disease Burden Initiative. Using the 2001 census, 7,597 geographic units, 4,433 (58·4%) of which were rural, were sampled for the 2004–13 SRS to represent the population of each state and union territory of India, ultimately with a sample of 6·7 million people that was equivalent to 0·7% of India's population. The SRS cause of death data were available for 2004–06, 2007–09, and 2010–13 year groups. 2005, 2008, and 2012 were used as midpoint years for these three time periods. The field investigation consists of continuous

enumeration of births and deaths in selected sample units by the enumerators and an independent survey every six months by SRS supervisors. The data obtained by these two independent functionaries are matched. The unmatched or partially matched events are re-verified in the field by a third party or jointly by the supervisor and the enumerator to get an unduplicated count of correct events. For every death occurring in the households, a verbal autopsy data was collected through a structured interview with a family member or close acquaintance of the deceased trained fieldworker by a trained field-worker. The verbal autopsy questionnaire was independently reviewed by two trained physicians to determine the probable cause of death based on the guidelines of ICD-10. In case of disagreement between the two physicians, the final ICD code was assigned by a third senior physician. The details of assignment of road injury as a cause of death in the SRS data have been reported previously.[2] Road injury deaths were identified based on translation of the open-ended narratives from the verbal autopsy tool into English from 14 local languages, and the modes of transportation were systematically extracted using a standardised data extraction tool and procedure. A substantial inter-rater agreement has been reported for assignment of road injury deaths and mode of transportation between the investigators and data extractors, and this method has been suggested to be robust in discerning between types of injury deaths.[3,4]

Building upon the previous Model Registration System of the Office of the Registrar General of India, data on causes of death in rural areas of India were collected by the Survey of Causes of Deaths (Rural) from 1980 to 1998 in a sample of villages of selected primary health centers using the verbal autopsy method.[5] In each state the number of primary health centres selected were based on the norm of atleast on unit per million of the population. Almost all the districts within the state were covered. A paramedical field agent from the primary health centre was designated with the task of interviewing the family of the decreased, and recording the symptoms, conditions, anatomical site and duration of illness using a structured questionnaire. A checklist of the non-medical causes of death based on ICD-9 was used. The probable cause of death was ascertained by applying the structured questionnaire to the symptoms and circumstances recorded. The primary health centre statistician was designated to do a half-yearly verification of the household list and the events reported by the field agent. The correctness of the cause of death assigned by the field agent was certified by the medical officer of the primary health centre. The survey design used was reasonably valid and fulfilled the design criteria for a good verbal autopsy system.[5] This survey provides reasonable data on the cause of death due to accidents and injuries, as these symptoms can easily be recognized by a lay person.[5] In 1999, Survey of Causes of Deaths (Rural) was merged with the SRS verbal autopsy cause of death data collection, covering both rural and urban areas.

The India Cause of Death Data Set Version 1.3 available from the Institute of Health Systems contains data from the Survey of Cause of Death (Rural) for the years 1980-1998, the Indian National List dataset of diseases (INL-9), and the Medical Certification of Cause of Death (MCCD) for the year 1991 to 1996. The data from the Survey of Cause of Death (Rural) for the years 1980-1995 were used from this source for estimating road injury deaths in the states of India.

The Indian Council of Medical Research Study on Causes of Death by Verbal Autopsy was carried out in five states of India namely, Assam, Bihar, Maharashtra, Rajasthan, and Tamil Nadu to assess probable causes of deaths in 2003.[6] A stratified multi-stage sampling design was adopted. Six-monthly survey was conducted in each state to collect information during the first and the second half of the year 2003. The reporting of deaths was supplemented by death reports from the health workers or private practitioners or prominent persons in the locality and list of deaths from the Municipal Corporation in urban area and Gram Panchayat in villages during a fixed reference period. After identifying the households, a close relative or caretaker or neighbour who attended the deceased person during the terminal phase preceding death was contacted for details information of the illness or events leading to death. The verbal autopsy method used for the data collection was exhaustive containing verbatim questions on symptoms, signs and modules. Based on the responses about the signs and symptoms preceding death, the cause of death was determined by physicians according to ICD-10 codes. For checking the completeness of deaths reported in the survey, estimated deaths based on sampling design by age, sex and place of residence were compared with those estimated in SRS for 2003 for each of the five selected states.

The International Network for the Demographic Evaluation of Populations and Their Health (INDEPTH) is a global network of research centers that conduct longitudinal health and demographic evaluation of populations in low- and middle-income countries including India.[7,8] The INDEPTH report provided data on causes of deaths for the states of Haryana and Maharashtra in 1992. Households in the surveillance area were visited regularly by lay field-workers, with a frequency varying from once per year to several times per year. All vital events were registered at each such visit, and any deaths recorded were followed up with

verbal autopsy interviews, usually undertaken by specially trained lay interviewers. The interpretation of the verbal autopsy data was done by local physicians, often more than one per case, in order to arrive at a consensus cause.

Public Health Foundation of India Cause of Death Estimation Study was done in all 38 districts of Bihar during 2011-2014.[9] This survey used a multi-stage stratified random sampling approach to obtain a sample of households representative of the state. All the households were enumerated and trained interviewers documented the age and sex of all the usual residents in each household during using the Open Development Kit software in hand-held tablets. Verbal autopsy interviews were conducted for the households that reported at least one death between 2012 to 2014 using the Population Health Metrics Research Consortium shortened verbal autopsy questionnaire.[10] A household member >18 years who was most aware of the context of death and/or illness preceding death was selected as the respondent for the interview. A direct question was asked to document if the deceased had suffered an injury/accident that led to death and type and intent of injury. This was followed by recording verbatim an open narrative of the death with the aim of documenting the context around the death. The cause of death was assigned using the validated SmartVA automated algorithm.[11]

Bias of categories of input data

Variation in data quality was addressed in the GBD study through a series of methods that include data standardisation and the redistribution of inappropriately coded deaths or “garbage codes” that were not possible causes of death, or that were not specific underlying causes of death, and had been entered as the underlying cause of death on death certificates. Undercounting or misassignment of deaths from road injuries is a known problem,[12-15] and the level and type of misassignment differs by location, age, and sex. The GBD study corrects for undercounting by first defining the "universe" of data of all deaths coded under the group's garbage codes or redistribution targets for each location, age, sex, and year. Correction of misassignment is accounted for in part by reassignment from Y26 (exposure to smoke, fire and flames, undetermined intent and equivalent code in ICD9) and X59 (exposure to unspecified factor) which were the biggest “intermediate causes” for injuries in the GBD Causes of Death framework. They were assigned to injuries but further redistributed to more specific injury causes using a regression method based on patterns of similar ICD codes. In addition, there were other ICD codes that may include road injuries deaths, such as undetermined intent injuries codes (Y10-Y34 in ICD-10 and E980-E988 in ICD-9) or exposure to unspecified factor (X59 in ICD-10; E887 in ICD-9), some intermediate causes of death that cannot be specific underlying causes of death (eg., septicaemia or peritonitis), or as poorly defined or unknown causes of mortality (R99). For distribution of intermediate causes, a regression was used between road injuries fractions and intermediate causes by age and sex in each location for each cause of injury. The same regressions were implemented for pedestrian injuries, cyclist injuries, motorcyclist injuries, motor vehicle occupant injuries and other road injuries. Based on scale up betas from these three regressions to one, redistribution of deaths coded to indeterminate causes to pedestrian injuries, cyclist injuries, motorcyclist injuries, motor vehicle occupant injuries and other road injuries were done. Distribution of garbage codes is explained in greater detail in a previous publication from the GBD 2017 study.[1] Using this approach in the Sample Registration System verbal autopsy data for India, 86.2% of the road injuries deaths were assigned directly, and 13.8% were assigned as road injuries deaths based on redistribution of garbage codes.

Mortality estimation

Formatting of data sources for the cause of death database has been described in detail elsewhere.[1] Mortality from road injuries was estimated using the standardised data from the GBD cause of death database and covariates using the cause of death ensemble model (CODEm) developed for the GBD study. Methods describing the CODEm approach have been described elsewhere.[1] In brief, ensemble modelling is a method where a large number of model specifications are systematically tested and reviewed based on their out-of-sample predictive validity. Models that perform best are subsequently incorporated into a weighted ensemble model with the highest weights assigned to models with the best out-of-sample prediction error. The CODEm model for road injuries generated estimates across all age groups. CODEm models estimate the individual cause-level mortality without taking into account the all-cause mortality. To ensure that all single causes add up to the all-cause mortality and that all sub-causes add up to the parent cause, an algorithm called “CodCorrect” is used. Details regarding the algorithm can be found elsewhere.[1]

As cause of death data in India are not available for all states for the entire duration from 1990 to 2017, GBD used covariates, which are variables that have an established association with the outcome of interest, to arrive at the best possible estimates of the cause of death. The influence of the various covariates is shown in the following graphs.

Males

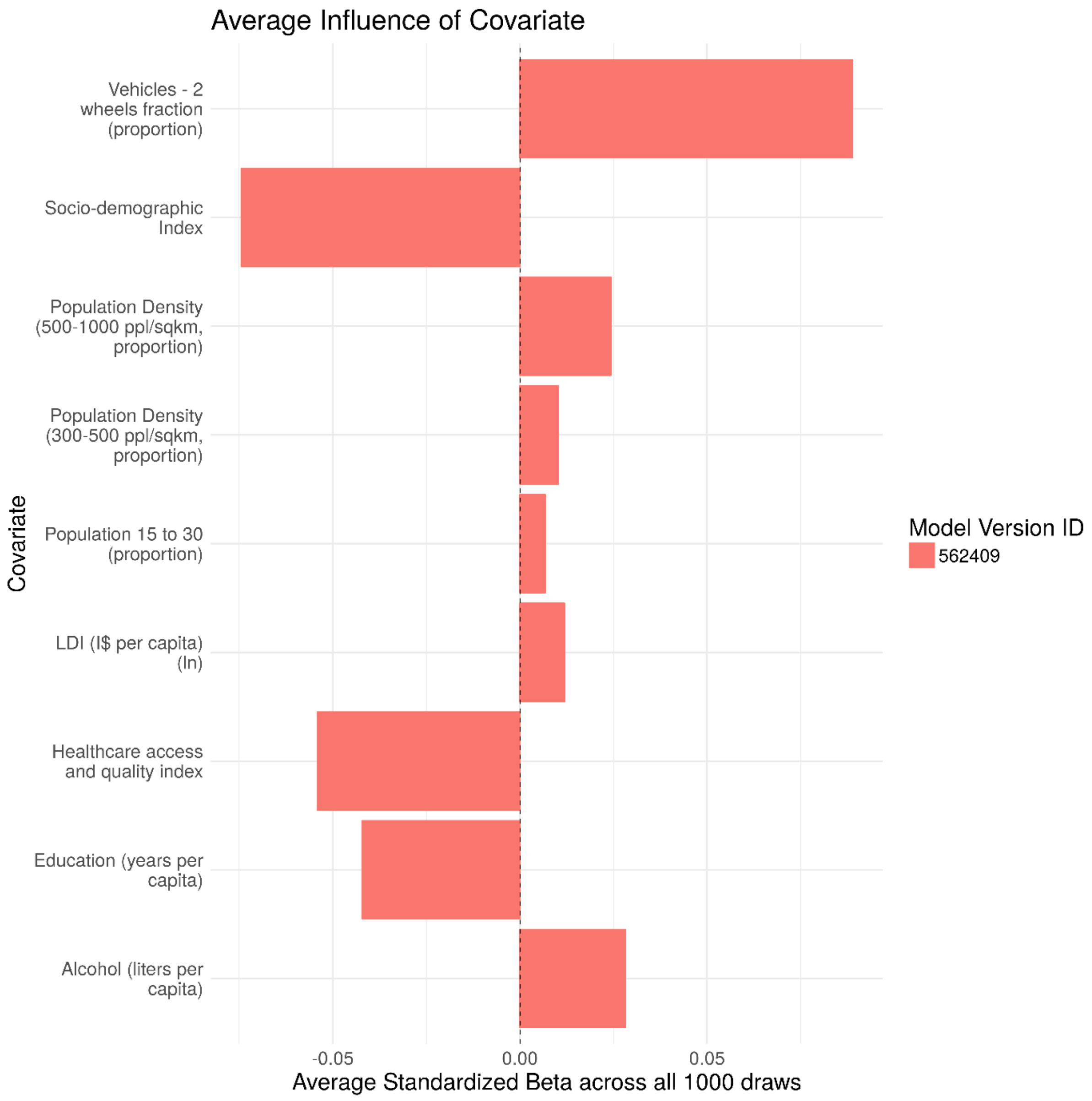

Females

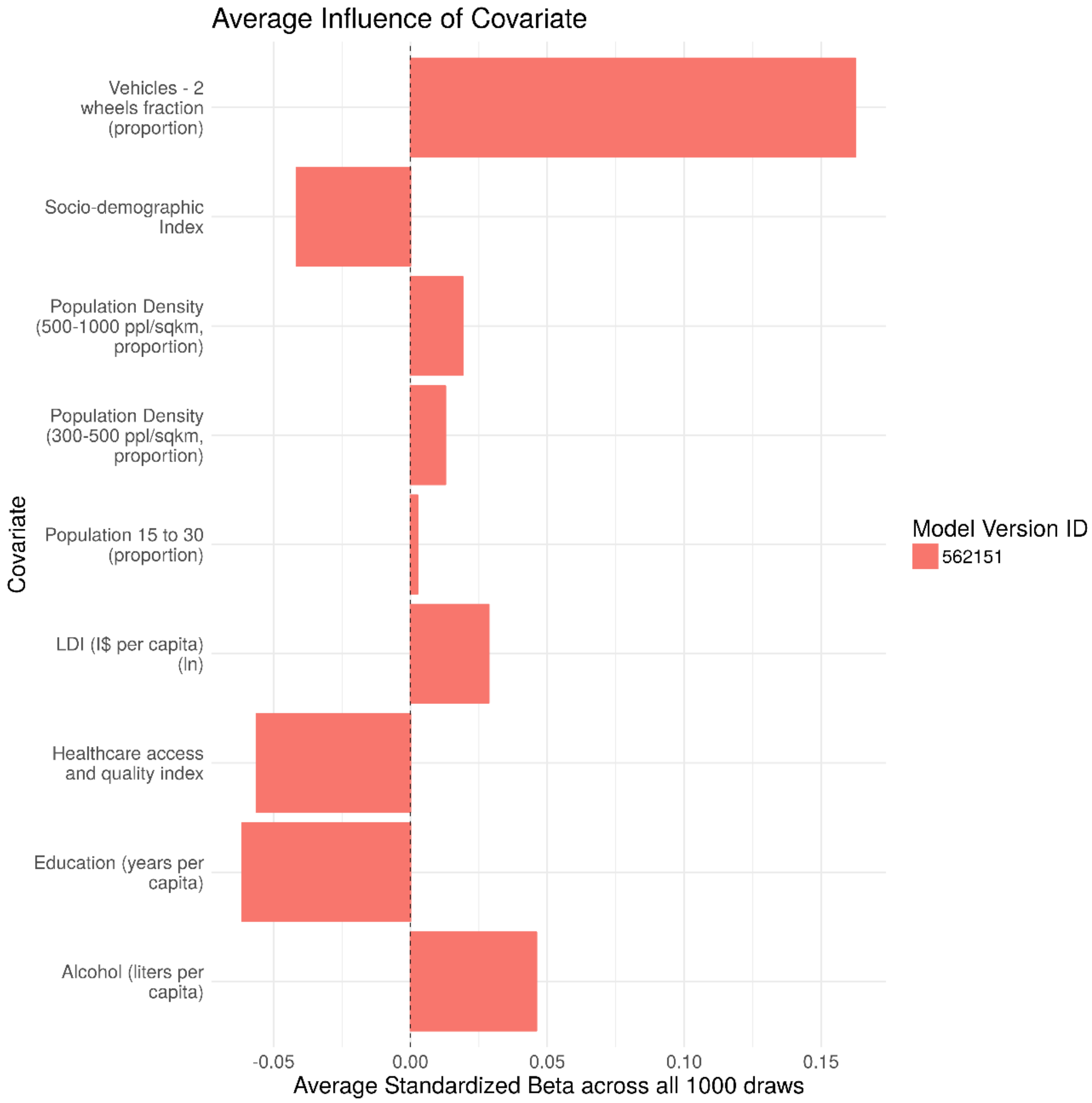

C. Uncertainty intervals

Point estimates for each quantity of interest were derived from the mean of the draws, while 95% uncertainty intervals (UIs) were derived from the 2.5th and 97.5th percentiles. Uncertainty in the estimation is attributable to sample size variability within data sources, different availability of data by age, sex, year, or location, and cause specific model specifications. We determined UIs for components of cause-specific estimation based on 1000 draws from the posterior distribution of cause specific mortality by age, sex, and location for each year included in the GBD 2017 analysis. In this way, uncertainty could be quantified and propagated into the final quantities of interest.

We included 95% uncertainty intervals (UIs) for each point estimate; results were considered statistically significant where the UI does not include zero. The age-standardised rates were based on the GBD global reference population, which is a time-invariant standard. Details of this calculation are available in the online appendix to the GBD 2017 Causes of Death publication.[1]

D. Projections to 2030

GBD 2017 produced projections for the health-related Sustainable Development Goal (SDG) indicators up to 2030 based on past trends, using a new advanced modelling framework.[5] The steps used to produce projections for the age standardised road injuries death rate are as follows.

The annual change from the previous year was first calculated from 1990 to 2017 using the logit of the prevalence for each year. The weight for each year was calculated using this formula:

$$weight_{year} = (t - 1990 + 1)^{\omega}$$

where ω is the weight function, the value of which denotes how much higher impact recent years would have compared with the past years when calculating the annual rate of change for the projection. To determine the appropriate value of ω for each indicator, an out-of-sample predictive validity test was done using data from 1990 to 2007 to predicted values for the years from 2008 to 2017. Assuming a range of values, in the increments of 0.25, from 0 to 10 for ω, the best predicted value for the period 2008 to 2017 was tested for each indicator. The final value for the weight function (ω) specific to each indicator for projection was chosen that minimised the root mean squared error in the 2008–2017 projections based on the 1990–2007 data. The weight function used for the road injuries death rate was 1.0.

The inverse of the weighted logit mean of the annualized rate of change from 1991 to 2017 was then applied to the years 2018 onward to estimate the age standardised road injuries death rate up to 2030, which takes into account the trends observed up to 2017.

References

1. GBD 2017 Causes of Death Collaborators. Global, regional and national age-sex specific mortality for 282 causes of death in 195 countries and territories,1980-2017: a systematic analysis for the Global Burden of Disease Study 2017. *Lancet* 2018, 392: 1736-1788.
2. Office of the Registrar General & Census Commissioner, Ministry of Home Affairs, Government of India. 2019. http://www.censusindia.gov.in/vital_statistics/SRS_Statistical_Report.html (accessed Oct 21, 2019).
3. Hsiao M, Malhotra A, Thakur JS, et al. Road traffic injury mortality and its mechanisms in India: nationally representative mortality survey of 1.1 million homes. *BMJ open* 2013; 3: e002621
4. Hsiao M, Morris SK, Bassani DG, Montgomery AL, Thakur JS, Jha P. Factors associated with physician agreement on verbal autopsy of over 11500 injury deaths in India. *PLoS One* 2012; 7: e30336.
5. Mahapatra P, Chalapati Rao P. Cause of death reporting systems in India: a performance analysis. *Natl Med J India* 2001; 14:154-62.
6. Indian Council of Medical Research. Study on Causes of Death by Verbal Autopsy in India. New Delhi, India; 2009. https://www.icmr.nic.in/sites/default/files/reports/Contents_NCD.pdf (accessed Oct 21, 2019).
7. Streatfield PK, Khan WA, Bhuiya A, Alam N, Sié A, Soura AB, et al. Cause-specific mortality in Africa and Asia: evidence from INDEPTH health and demographic surveillance system sites. *Glob Health Action* 2014;7.
8. Herbst K, Juvekar S, Bhattacharjee T, Bangha M, Patharia N, Tei T, et al. The INDEPTH Data Repository. *J Empir Res Hum Res Ethics* 2015; 10:324–33.
9. Dandona R, Kumar GA, Kharyal A, George S, Akbar M, Dandona L. Mortality due to snakebite and other venomous animals in the Indian state of Bihar: Findings from a representative mortality study. *PLoS One*. 2018;13: e0198900.
10. Serina P, Riley I, Stewart A, Flaxman AD, Lozano R, Mooney MD, et al. A shortened verbal autopsy instrument for use in routine mortality surveillance systems. *BMC Med*. 2015;13: 302.
11. Serina P, Riley I, Stewart A, James SL, Flaxman AD, Lozano R, et al. Improving performance of the Tariff Method for assigning causes of death to verbal autopsies. *BMC Med*. 2015;13: 291.
12. Dandona R, Kumar GA, Ameer MA, Reddy GB, Dandona L. Under-reporting of road traffic injuries to the police: results from two data sources in urban India. *Inj Prev*. 2008; 14:360-5.
13. Raban MZ, Dandona L, Dandona R. The quality of police data on RTC fatalities in India. *Inj Prev*. 2014; 20:293-301.
14. Bhalla K, Khurana N, Bose D, Navaratne KV, Tiwari G, Mohan D. Official government statistics of road traffic deaths in India under-represent pedestrians and motorised two wheeler riders. *Inj Prev*. 2017; 23: 1-7.

15. Foreman KJ, Marquez N, Dolgert A, et al. Forecasting life expectancy, years of life lost, all-cause and cause-specific mortality for 250 causes of death: reference and alternative scenarios for 2016–2040 for 195 countries and territories. *Lancet* 2018; 392: 2052–90.

2. Grouping of the states of India based on SDI, 2017

State group (population in 2017)	States of India	SDI in 2017
**Low SDI states (675 million)**	Bihar	0.43
	Madhya Pradesh	0.49
	Jharkhand	0.49
	Uttar Pradesh	0.49
	Rajasthan	0.49
	Chhattisgarh	0.51
	Odisha	0.52
	Assam	0.53
**Middle SDI states (387 million)**	Andhra Pradesh	0.54
	West Bengal	0.54
	Tripura	0.54
	Arunachal Pradesh	0.56
	Meghalaya	0.56
	Karnataka	0.57
	Telangana	0.58
	Gujarat	0.58
	Manipur	0.59
	Jammu and Kashmir*	0.59
	Haryana	0.60
**High SDI states (318 million)**	Uttarakhand	0.61
	Tamil Nadu	0.62
	Mizoram	0.62
	Maharashtra	0.62
	Punjab	0.62
	Sikkim	0.63
	Nagaland	0.63
	Himachal Pradesh	0.63
	Union territories other than Delhi	0.65
	Kerala	0.66
	Delhi	0.72
	Goa	0.74

SDI=Socio-demographic Index.
SDI as computed by GBD in 2017 as described elsewhere (*Lancet* 2018; 392: 1995-2051).
*The state of Jammu and Kashmir was divided into two union territories in August 2019; as we are reporting findings up to 2017, we report findings for the state of Jammu and Kashmir.

3. Proportion of road injury deaths among the total deaths in each age group in males and females in India, 1990 and 2017

Age groups (years)	Proportion of road injury deaths among the total deaths in males (95% uncertainty interval)		Proportion of road injury deaths among total deaths in females (95% uncertainty interval)	
	**1990**	**2017**	**1990**	**2017**
All ages	2.3 (2.1-2.5)	3.2 (3.0-3.4)	0.8 (0.8-0.9)	1.1 (0.9-1.2)
Under 5	0.4 (0.3-0.5)	0.4 (0.3-0.6)	0.3 (0.3-0.4)	0.4 (0.3-0.6)
5 to 9	3.6 (2.8-4.5)	4.5 (3.3-6.6)	2.2 (2.2-2.8)	3.5 (2.7-4.4)
10 to 14	4.8 (3.8-5.9)	6.5 (5.3-7.9)	2.1 (2.1-2.6)	3.0 (2.5-3.8)
15 to 19	11.0 (9.5-12.6)	15.8 (13.3-20.3)	2.1 (2.1-2.5)	3.1 (2.7-4.1)
20 to 24	13.7 (12.3-15.1)	19.6 (18.0-21.3)	2.1 (2.1-2.4)	3.5 (3.0-4.1)
25 to 29	12.2 (11.0-13.4)	17.0 (15.7-18.6)	2.4 (2.4-2.6)	3.4 (3.0-3.9)
30 to 34	9.4 (8.5-10.3)	13.1 (11.7-14.2)	2.3 (2.3-2.6)	3.5 (3.1-3.9)
35 to 39	7.8 (7.1-8.6)	10.7 (9.4-11.7)	2.4 (2.4-2.6)	3.5 (3.0-3.9)
40 to 44	5.4 (5.0-5.9)	7.5 (6.6-8.2)	1.9 (1.9-2.1)	2.8 (2.1-3.1)
45 to 49	4.0 (3.7-4.4)	5.6 (4.8-6.3)	1.5 (1.5-1.7)	2.2 (1.7-2.5)
50 to 54	2.9 (2.6-3.1)	3.9 (3.5-4.3)	1.3 (1.3-1.4)	1.9 (1.1-2.1)
55 to 59	2.0 (1.8-2.1)	2.6 (1.9-3.0)	1.0 (1.0-1.1)	1.4 (1.1-1.6)
60 to 64	1.4 (1.2-1.5)	1.9 (1.6-2.1)	0.8 (0.8-0.9)	1.3 (1.0-1.4)
65 to 69	1.0 (0.9-1.1)	1.4 (1.3-1.6)	0.6 (0.6-0.6)	0.8 (0.7-0.9)
70 to 74	0.8 (0.7-0.8)	1.0 (0.9-1.1)	0.4 (0.4-0.5)	0.6 (0.5-0.7)
75 to 79	0.6 (0.6-0.7)	0.9 (0.8-1.0)	0.3 (0.3-0.4)	0.5 (0.4-0.5)
≥80	0.4 (0.4-0.5)	0.6 (0.5-0.7)	0.2 (0.2-0.3)	0.3 (0.3-0.4)

4. Road injury death rate by type of road users in the states of India, 2017

Both sexes combined

	Crude death rate per 100,000 (95% uncertainty interval), 2017					Age-standardised death rate per 100,000 (95% uncertainty interval), 2017				
States of India	Overall	Pedestrian	Motor cyclist	Motor vehicle occupant	Cyclist	Overall	Pedestrian	Motor cyclist	Motor vehicle occupant	Cyclist
**India**	**15.9 (14.6-16.7)**	**5.6 (4.8-6.2)**	**4.9 (3.9-3.4)**	**4.2 (3.7-5.2)**	**1.1 (0.8-1.3)**	**17.2 (15.7-18.1)**	**6.4 (5.5-7.1)**	**4.9 (3.9-5.4)**	**4.5 (3.9-5.6)**	**1.2 (0.9-1.4)**
**Low SDI states**	**15.9 (14.8-16.9)**	**5.5 (4.9-6.1)**	**1.2 (0.9-1.3)**	**4.1 (3.7-5.4)**	**5.5 (4.9-6.1)**	**18.6 (17.1-19.9)**	**6.9 (6.1-7.7)**	**5.3 (4.4-5.9)**	**4.8 (4.2-6.3)**	**1.5 (0.9-1.8)**
Bihar	11.4 (10.2-12.9)	5.6 (4.5-6.4)	2.5 (2.0-2.3)	2.7 (2.2-3.9)	0.6 (0.4-0.7)	14.4 (12.8-16.1)	7.4 (5.9-8.4)	2.9 (2.3-3.7)	3.3 (2.7-4.8)	0.7 (0.5-0.9)
Madhya Pradesh	16.1 (14.3-18.2)	4.9 (4.2-5.6)	5.0 (4.0-4.0)	4.9 (4.2-5.8)	1.2 (0.8-1.5)	18.5 (16.3-20.9)	6.1 (5.2-7.0)	5.3 (4.2-6.3)	5.5 (4.7-6.5)	1.4 (1.0-1.7)
Jharkhand	16.9 (15.2-18.8)	7.0 (6.3-7.9)	3.0 (2.4-2.3)	5.4 (4.5-6.2)	1.5 (0.6-2.1)	19.9 (18.0-22.0)	8.8 (7.8-9.9)	3.1 (2.6-4.6)	6.2 (5.1-7.1)	1.7 (0.7-2.4)
Uttar Pradesh	18.1 (16.2-20.3)	6.5 (5.5-7.6)	5.8 (4.4-4.9)	4.1 (3.4-6.0)	1.6 (0.8-2.0)	21.6 (19.2-24.4)	8.3 (7.0-9.7)	6.5 (4.9-7.6)	4.8 (3.9-7.0)	1.9 (1.0-2.5)
Rajasthan	17.0 (14.8-19.3)	4.9 (4.0-5.8)	6.5 (4.2-4.8)	4.5 (3.8-5.7)	0.9 (0.7-1.1)	19.8 (17.2-22.5)	6.2 (5.1-7.4)	7.1 (4.6-8.6)	5.3 (4.3-6.7)	1.1 (0.9-1.3)
Chhattisgarh	19.0 (15.6-21.7)	4.8 (3.9-6.1)	7.0 (5.2-5.6)	5.3 (4.0-7.7)	1.7 (1.0-2.1)	21.1 (17.3-24.0)	5.7 (4.6-7.1)	7.3 (5.3-8.9)	5.9 (4.5-8.4)	1.9 (1.1-2.4)
Odisha	14.3 (12.8-15.8)	3.3 (2.8-3.9)	4.4 (3.8-3.1)	4.6 (4.0-6.0)	1.8 (1.0-2.3)	14.8 (13.3-16.4)	3.6 (3.1-4.3)	4.3 (3.7-5.0)	4.8 (4.2-6.2)	1.9 (1.1-2.4)
Assam	11.9 (10.7-13.3)	5.6 (4.5-6.4)	3.1 (2.5-2.0)	3.7 (3.1-5.0)	1.2 (0.7-1.6)	13.6 (12.2-15.2)	4.8 (4.0-5.4)	3.1 (2.6-4.1)	4.1 (3.5-5.7)	1.5 (0.8-1.8)
**Middle SDI states**	**14.9 (13.4-16.0)**	**5.5 (4.9-6.1)**	**4.3 (3.6-4.8)**	**4.3 (3.7-5.0)**	**0.9 (0.7-1.0)**	**15.3 (13.7-16.5)**	**5.9 (5.0-6.6)**	**4.1 (3.4-4.6)**	**4.3 (3.7-5.0)**	**0.9 (0.7-1.0)**
Andhra Pradesh	15.9 (12.6-19.9)	3.9 (3.1-4.4)	4.3 (3.2-3.6)	4.0 (3.0-5.4)	0.6 (0.4-1.0)	15.8 (12.4-19.8)	7.0 (5.4-8.7)	4.0 (3.0-5.2)	4.0 (3.0-5.3)	0.6 (0.4-1.0)
West Bengal	12.2 (10.7-13.8)	6.7 (5.3-8.4)	2.0 (1.6-1.3)	3.1 (2.6-4.0)	1.1 (0.6-1.4)	12.6 (11.1-14.2)	6.3 (5.1-7.4)	1.9 (1.5-3.1)	3.1 (2.6-4.0)	1.2 (0.7-1.5)
Tripura	12.8 (10.2-16.2)	5.9 (4.6-6.8)	1.8 (1.1-1.5)	4.8 (3.7-6.3)	1.3 (0.8-1.8)	13.3 (10.8-16.6)	5.3 (4.3-6.4)	1.7 (1.0-4.3)	4.8 (3.8-6.2)	1.4 (0.8-1.8)
Arunachal Pradesh	10.6 (8.3-14.0)	4.9 (3.8-6.0)	3.0 (2.1-2.6)	4.9 (3.7-6.4)	0.7 (0.5-1.0)	14.2 (11.2-18.1)	3.2 (2.3-4.7)	3.5 (2.5-5.2)	6.4 (4.8-8.1)	1.0 (0.8-1.5)
Meghalaya	7.8 (6.3-9.9)	2.0 (1.5-3.1)	1.0 (0.7-0.9)	4.0 (2.5-5.3)	0.5 (0.3-0.7)	10.0 (8.2-12.4)	3.2 (2.6-4.0)	1.1 (0.7-3.2)	5.0 (3.3-6.4)	0.6 (0.5-1.0)
Karnataka	17.0 (13.5-19.3)	2.3 (1.8-2.8)	6.1 (4.5-4.6)	5.0 (3.8-6.5)	0.7 (0.4-1.2)	16.9 (13.6-19.2)	5.4 (4.1-6.6)	5.7 (4.1-7.0)	5.0 (3.8-6.4)	0.7 (0.5-1.3)
Telangana	14.1 (11.1-17.7)	5.0 (3.8-6.2)	3.9 (2.8-2.2)	3.6 (2.7-4.8)	0.6 (0.4-0.8)	14.4 (11.4-17.9)	6.3 (4.9-7.7)	3.7 (2.7-4.8)	3.6 (2.8-4.8)	0.6 (0.4-0.9)
Gujarat	13.5 (12.1-15.0)	5.8 (4.6-7.3)	4.8 (3.6-3.6)	4.8 (4.1-5.4)	0.6 (0.5-1.0)	14.1 (12.7-15.5)	3.6 (3.1-4.2)	4.7 (3.5-5.4)	5.0 (4.3-5.7)	0.7 (0.5-1.1)
Manipur	18.4 (14.8-23.0)	3.3 (2.8-3.8)	4.2 (3.1-3.4)	5.0 (3.7-6.5)	0.7 (0.5-1.1)	21.2 (17.0-26.0)	10.4 (8.3-12.9)	4.3 (3.2-5.5)	5.5 (4.1-7.1)	0.8 (0.6-1.2)
Jammu and Kashmir*	20.0 (17.2-22.8)	8.3 (6.6-10.5)	4.5 (3.6-3.5)	6.8 (4.0-8.2)	0.6 (0.5-1.0)	21.9 (18.7-24.9)	9.2 (7.5-11.5)	4.6 (3.6-5.6)	7.2 (4.4-8.8)	0.7 (0.5-1.1)
Haryana	20.5 (17.9-22.8)	8.0 (6.5-10.1)	7.6 (4.9-4.0)	5.3 (4.2-6.8)	1.6 (1.1-2.0)	21.9 (18.9-24.4)	6.7 (5.4-8.3)	7.5 (4.8-9.0)	5.6 (4.6-7.2)	1.8 (1.2-2.2)
**High SDI states**	**14.9 (13.4-16.0)**	**5.5 (4.9-6.1)**	**5.7 (3.8-6.5)**	**4.2 (3.5-5.5)**	**1.2 (0.9-1.3)**	**16.6 (14.7-17.9)**	**5.9 (5.0-6.9)**	**5.3 (3.6-6.1)**	**4.1 (3.5-5.3)**	**1.1 (0.9-1.3)**
Uttarakhand	24.8 (21.2-28.4)	7.6 (6.1-10.4)	8.1 (5.6-5.0)	7.1 (5.1-8.3)	2.0 (1.3-2.5)	26.3 (22.5-29.8)	8.6 (6.8-11.7)	8.0 (5.5-9.9)	7.4 (5.4-8.7)	2.1 (1.4-2.7)
Tamil Nadu	20.8 (18.4-23.0)	7.5 (6.3-9.2)	6.6 (4.2-4.8)	4.9 (4.0-6.3)	1.7 (1.0-2.0)	20.0 (17.7-22.1)	7.6 (6.4-9.2)	6.0 (3.9-7.1)	4.7 (3.9-6.0)	1.6 (0.9-1.9)
Mizoram	14.0 (11.1-17.4)	2.0 (1.5-2.8)	6.5 (4.3-4.5)	4.7 (3.6-6.5)	0.6 (0.4-1.4)	15.9 (12.7-19.5)	2.6 (2.0-3.5)	7.0 (4.4-9.2)	5.4 (4.1-7.5)	0.8 (0.5-1.7)
Maharashtra	13.6 (11.9-15.1)	4.5 (3.8-5.2)	5.0 (3.4-3.0)	3.4 (2.8-4.8)	0.6 (0.4-1.1)	13.6 (11.8-15.1)	4.7 (3.9-5.5)	4.8 (3.2-5.7)	3.4 (2.8-4.8)	0.6 (0.4-1.1)
Punjab	23.9 (20.8-26.9)	7.0 (5.9-8.8)	8.6 (4.8-4.3)	5.8 (4.7-7.6)	2.4 (1.0-3.0)	22.9 (20.0-25.7)	7.0 (5.9-8.6)	8.0 (4.5-9.6)	5.5 (4.6-7.2)	2.3 (1.0-2.9)
Sikkim	10.8 (8.8-13.4)	3.4 (2.7-4.2)	1.7 (1.1-1.7)	5.3 (3.7-6.7)	0.4 (0.3-1.0)	11.7 (9.4-14.4)	4.0 (3.1-5.0)	1.6 (1.0-3.5)	5.5 (3.9-6.8)	0.5 (0.3-1.2)
Nagaland	11.2 (8.6-14.2)	2.5 (1.8-3.8)	2.9 (2.1-2.9)	5.4 (3.5-7.2)	0.4 (0.2-1.0)	14.1 (10.8-17.7)	3.4 (2.4-5.2)	3.1 (2.3-4.2)	6.9 (4.3-9.1)	0.5 (0.3-1.3)
Himachal Pradesh	18.4 (14.3-21.4)	4.0 (2.9-5.8)	4.9 (3.3-3.9)	8.3 (5.3-10.3)	1.1 (0.8-1.7)	17.5 (13.8-20.4)	3.9 (2.9-5.7)	4.5 (3.1-6.4)	7.9 (5.1-9.7)	1.1 (0.8-1.6)
Union territories other than Delhi	13.3 (10.5-16.4)	5.2 (3.9-6.7)	4.6 (3.0-3.9)	2.7 (2.0-4.2)	0.8 (0.6-1.0)	14.0 (11.0-17.1)	6.0 (4.7-7.6)	4.3 (2.8-5.6)	2.8 (2.0-4.3)	0.8 (0.6-1.1)
Kerala	15.9 (13.3-18.1)	6.9 (5.6-8.3)	4.9 (2.8-2.0)	3.1 (2.3-4.4)	1.0 (0.8-1.3)	14.0 (11.7-15.8)	5.9 (4.8-7.1)	4.4 (2.6-5.4)	2.7 (2.1-3.9)	0.8 (0.6-1.1)
Delhi	11.0 (9.1-12.7)	3.8 (2.9-4.7)	2.9 (2.1-2.6)	3.3 (2.6-4.0)	0.9 (0.7-1.2)	11.8 (9.8-13.6)	4.4 (3.3-5.4)	2.8 (2.0-3.5)	3.6 (2.7-4.3)	1.0 (0.7-1.2)
Goa	12.3 (9.5-15.3)	4.8 (3.6-6.2)	4.5 (2.6-2.9)	2.5 (1.8-3.9)	0.4 (0.3-1.1)	11.1 (8.7-13.8)	4.4 (3.4-5.6)	4.0 (2.3-5.3)	2.2 (1.6-3.5)	0.4 (0.3-1.0)

Males

	Crude death rate per 100,000 (95% uncertainty interval), 2017					Age-standardised death rate per 100,000 (95% uncertainty interval), 2017				
States of India	Overall	Pedestrian	Motor cyclist	Motor vehicle occupant	Cyclist	Overall	Pedestrian	Motor cyclist	Motor vehicle occupant	Cyclist
**India**	**23.7 (21.8-25.2)**	**7.6 (6.4-8.7)**	**8.2 (6.4-9.1)**	**5.8 (5.2-7.7)**	**1.9 (1.3-2.2)**	**25.7 (23.5-27.4)**	**8.9 (7.5-10.2)**	**8.3 (6.4-9.3)**	**6.2 (5.4-8.2)**	**2.1 (1.4-2.5)**
**Low SDI states**	**23.3 (21.3-25.2)**	**7.4 (6.4-8.5)**	**7.9 (6.4-8.9)**	**5.7 (5.0-7.8)**	**2.2 (1.2-2.6)**	**27.3 (24.6-29.6)**	**9.5 (8.0-10.8)**	**8.6 (6.9-9.7)**	**6.5 (5.6-8.9)**	**2.6 (1.4-3.1)**
Bihar	15.3 (13.2-17.9)	7.3 (5.4-8.7)	3.8 (2.8-5.1)	3.3 (2.5-5.6)	1.0 (0.7-1.3)	18.9 (16.0-21.9)	9.5 (7.1-11.3)	4.2 (3.1-5.6)	3.9 (3.0-6.6)	1.2 (0.8-1.5)
Madhya Pradesh	24.3 (21.1-27.9)	6.5 (5.4-7.8)	8.3 (6.5-10.1)	7.4 (6.1-9.1)	1.9 (1.3-2.4)	27.6 (23.9-31.7)	8.1 (6.7-9.7)	8.7 (6.8-10.5)	8.2 (6.7-10.1)	2.3 (1.5-2.9)
Jharkhand	22.5 (19.6-26.6)	8.1 (6.9-9.4)	5.2 (4.3-6.9)	6.3 (5.1-8.2)	2.7 (1.1-4.0)	26.0 (22.8-30.2)	10.1 (8.7-11.8)	5.5 (4.5-7.2)	7.0 (5.6-9.0)	3.2 (1.3-4.6)
Uttar Pradesh	26.9 (23.3-30.7)	8.9 (7.3-10.8)	9.4 (7.0-11.3)	5.8 (4.6-8.9)	2.6 (1.3-3.4)	32.3 (27.7-37.2)	11.7 (9.5-14.2)	10.4 (7.7-12.6)	6.7 (5.3-10.4)	3.3 (1.6-4.3)
Rajasthan	25.8 (21.7-30.0)	6.6 (5.3-8.3)	10.6 (6.7-13.0)	6.8 (5.5-8.7)	1.5 (1.2-1.8)	30.4 (25.4-35.4)	8.7 (6.9-10.8)	11.6 (7.3-14.3)	7.9 (6.3-10.0)	1.9 (1.4-2.3)
Chhattisgarh	28.5 (22.6-33.3)	7.3 (5.9-9.2)	11.9 (8.3-14.8)	5.9 (4.1-11.2)	3.0 (1.6-3.9)	32.6 (25.6-37.7)	9.4 (7.5-11.8)	12.4 (8.6-15.4)	6.6 (4.5-12.4)	3.6 (1.9-4.6)
Odisha	21.6 (19.1-24.3)	4.4 (3.5-5.3)	7.8 (6.6-9.1)	6.1 (4.9-9.0)	3.0 (1.5-4.0)	22.4 (19.8-25.2)	4.9 (3.9-6.0)	7.8 (6.5-9.0)	6.2 (5.1-9.2)	3.2 (1.5-4.1)
Assam	18.6 (16.4-21.1)	5.8 (4.5-6.8)	5.4 (4.4-7.0)	5.3 (4.3-7.5)	2.1 (1.1-2.7)	21.0 (18.5-23.5)	7.2 (5.8-8.4)	5.4 (4.5-7.1)	5.8 (4.7-8.2)	2.4 (1.3-3.1)
**Middle SDI states**	**22.4 (19.9-24.3)**	**7.4 (6.1-8.5)**	**7.4 (6.0-8.3)**	**6.0 (5.2-7.5)**	**1.5 (1.3-1.8)**	**23.1 (20.5-25.2)**	**8.3 (6.7-9.4)**	**7.1 (5.7-8.0)**	**6.1 (5.2-7.5)**	**1.5 (1.3-1.8)**
Andhra Pradesh	24.3 (18.8-30.7)	9.1 (6.8-11.6)	7.9 (5.7-10.3)	5.9 (4.3-8.4)	1.1 (0.8-1.8)	24.5 (18.9-30.9)	9.8 (7.3-12.3)	7.4 (5.4-9.7)	5.8 (4.2-8.2)	1.2 (0.8-1.8)
West Bengal	18.5 (15.8-21.4)	8.7 (6.7-10.3)	3.3 (2.4-5.7)	4.5 (3.6-6.3)	1.9 (1.0-2.5)	18.9 (16.2-21.8)	9.4 (7.3-11.1)	3.1 (2.3-5.3)	4.4 (3.5-6.1)	1.9 (1.0-2.5)
Tripura	20.2 (15.9-26.3)	7.1 (5.5-8.9)	3.2 (1.9-7.8)	7.6 (5.7-10.2)	2.2 (1.3-3.1)	21.2 (17.0-26.7)	8.0 (6.4-9.7)	3.0 (1.8-7.4)	7.6 (5.8-10.0)	2.4(1.4-3.2)
Arunachal Pradesh	16.9 (13.1-22.7)	3.7 (2.7-5.6)	5.0 (3.5-7.7)	7.1 (5.2-9.5)	1.1 (0.8-1.7)	22.3 (17.3-28.7)	6.1 (4.4-8.8)	5.7 (4.0-8.5)	8.9 (6.6-11.4)	1.6 (1.1-2.3)
Meghalaya	11.5 (9.1-14.9)	3.0 (2.1-3.8)	1.9 (1.2-5.0)	5.8 (3.5-8.1)	0.8 (0.5-1.3)	14.5 (11.7-18.2)	4.4 (3.2-5.4)	2.0 (1.3-5.6)	7.0 (4.5-9.4)	1.0 (0.8-1.8)
Karnataka	26.7 (21.0-31.0)	7.3 (5.5-9.4)	10.9 (7.6-13.7)	7.1 (5.3-9.9)	1.2 (0.8-2.3)	26.8 (21.0-31.0)	8.1 (6.1-10.2)	10.2 (7.0-12.8)	7.1 (5.3-9.7)	1.3 (0.8-2.3)
Telangana	21.5 (17.0-27.3)	7.8 (6.0-10.1)	7.2 (4.9-9.5)	5.3 (4.0-7.2)	1.0 (0.7-1.6)	22.1 (17.5-27.8)	8.6 (6.7-11.0)	6.8 (4.7-9.0)	5.3 (4.0-7.1)	1.1 (0.8-1.6)
Gujarat	18.8 (16.4-21.5)	4.1 (3.4-4.8)	7.6 (5.6-9.1)	6.0 (5.0-7.2)	1.0 (0.8-1.7)	19.9 (17.3-22.7)	4.8 (4.0-5.6)	7.5 (5.5-9.0)	6.3 (5.2-7.5)	1.1 (0.9-1.9)
Manipur	23.3 (18.5-29.5)	9.2 (7.0-11.8)	6.0 (4.5-8.1)	6.8 (5.0-8.8)	1.2 (0.9-2.0)	26.6 (21.1-32.7)	11.6 (9.0-14.6)	6.1 (4.5-8.1)	7.4 (5.5-9.4)	1.4 (1.0-2.3)
Jammu and Kashmir*	31.0 (26.3-36.1)	11.2 (8.8-15.2)	7.8 (6.0-9.6)	10.7 (5.7-13.5)	1.1 (0.9-1.8)	33.7 (28.5-39.0)	13.0 (10.3-17.3)	7.9 (5.9-9.6)	11.4 (6.0-14.2)	1.3 (1.0-2.0)
Haryana	29.5 (25.6-33.6)	6.3 (5.0-8.4)	13.2 (8.1-15.8)	7.2 (5.7-10.3)	2.6 (1.5-3.3)	31.6 (27.3-36.0)	7.5 (6.0-9.8)	13.2 (8.0-15.9)	7.7 (6.1-10.9)	3.0 (1.7-3.7)
**High SDI states**	**26.1 (23.4-28.4)**	**8.2 (6.6-10.1)**	**9.8 (6.5-11.2)**	**6.0 (5.0-8.2)**	**2.1 (1.5-2.4)**	**25.8 (23.1-28.0)**	**8.6 (6.9-10.5)**	**9.2 (6.1-10.6)**	**5.9 (4.9-7.9)**	**2.0 (1.5-2.4)**
Uttarakhand	39.7 (32.7-46.6)	11.2 (8.4-16.0)	14.2 (9.7-18.0)	10.7 (7.4-13.2)	3.4 (1.9-4.4)	42.3 (35.0-49.2)	12.9 (9.6-18.1)	14.1 (9.5-17.8)	11.2 (7.8-13.8)	3.8 (2.1-4.9)
Tamil Nadu	33.2 (29.1-37.4)	10.8 (8.5-14.2)	11.8 (7.5-14.1)	7.3 (5.7-10.2)	3.1 (1.7-3.9)	32.2 (28.2-36.1)	11.1 (8.9-14.5)	10.9 (6.9-13.0)	7.0 (5.5-9.7)	3.0 (1.6-3.7)
Mizoram	22.8 (18.2-28.6)	3.3 (2.5-4.7)	10.0 (7.3-13.0)	8.3 (6.2-10.7)	1.1 (0.7-2.6)	26.5 (21.2-32.5)	4.5 (3.4-6.3)	10.6 (7.7-13.7)	9.6 (7.2-12.3)	1.4 (1.0-3.2)
Maharashtra	20.2 (17.6-22.9)	6.0 (4.9-7.1)	8.6 (5.6-10.4)	4.6 (3.7-7.0)	0.8 (0.6-1.8)	20.1 (17.5-22.9)	6.4 (5.2-7.5)	8.1 (5.3-9.9)	4.6 (3.7-6.9)	0.9 (0.6-1.8)
Punjab	36.0 (31.0-41.0)	10.0 (8.1-12.9)	13.2 (7.9-16.2)	8.4 (6.7-11.1)	4.3 (1.7-5.5)	34.8 (30.1-39.6)	10.0 (8.2-12.9)	12.3 (7.4-15.1)	8.1 (6.5-10.7)	4.2 (1.7-5.4)
Sikkim	16.7 (13.4-20.8)	4.6 (3.5-5.8)	3.1 (2.0-6.4)	8.2 (5.4-10.5)	0.7 (0.4-1.9)	17.9 (14.1-22.0)	5.5 (4.2-6.9)	3.0 (1.9-6.1)	8.4 (5.7-10.6)	0.8 (0.5-2.2)
Nagaland	14.0 (10.8-17.8)	3.4 (2.4-4.5)	4.9 (3.6-6.3)	5.1 (3.8-6.7)	0.5 (0.2-1.5)	17.2 (13.3-21.6)	4.9 (3.5-6.4)	5.3 (3.9-6.8)	6.2 (4.6-8.0)	0.6 (0.3-2.0)
Himachal Pradesh	30.4 (22.9-36.0)	6.5 (4.5-9.5)	9.0 (6.1-12.7)	12.9 (7.3-16.6)	1.8 (1.2-2.8)	29.6 (22.4-35.0)	6.7 (4.7-9.6)	8.5 (5.7-12.0)	12.5 (7.2-16.1)	1.8 (1.2-2.7)
Union territories other than Delhi	18.4 (14.3-22.9)	6.0 (4.3-8.1)	6.9 (4.8-8.8)	4.0 (2.8-6.2)	1.4 (1.0-1.9)	19.6 (15.1-24.3)	7.3 (5.4-9.7)	6.5 (4.4-8.3)	4.1 (2.9-6.4)	1.6 (1.1-2.1)
Kerala	25.2 (20.4-29.5)	10.4 (7.9-13.1)	8.7 (5.2-10.8)	4.1 (3.0-6.8)	1.9 (1.4-2.5)	23.5 (19.1-27.4)	9.7 (7.3-12.2)	8.1 (5.0-10.2)	3.8 (2.7-6.3)	1.7 (1.3-2.2)
Delhi	16.3 (13.2-19.5)	5.3 (4.0-6.9)	4.9 (3.6-6.3)	4.4 (3.2-5.7)	1.6 (1.1-2.0)	17.5 (14.1-20.7)	6.3 (4.7-8.2)	4.7 (3.4-6.0)	4.5 (3.3-5.8)	1.7 (1.2-2.2)
Goa	18.5 (14.1-23.2)	6.7 (4.8-8.8)	7.6 (4.5-10.0)	3.4 (2.4-5.8)	0.8 (0.5-2.1)	17.5 (13.5-21.8)	6.7 (4.9-8.7)	6.8 (4.0-9.0)	3.2 (2.3-5.4)	0.7 (0.4-1.9)

Females

States of India	Crude death rate per 100,000 (95% uncertainty interval), 2017					Age-standardised death rate per 100,000 (95% uncertainty interval), 2017				
	Overall	Pedestrian	Motor cyclist	Motor vehicle occupant	Cyclist	Overall	Pedestrian	Motor cyclist	Motor vehicle occupant	Cyclist
**India**	**7.6 (6.5-8.1)**	**3.4 (2.9-3.8)**	**1.4 (1.1-1.7)**	**2.4 (2.0-2.9)**	**0.3 (0.2-0.3)**	**8.5 (7.2-9.1)**	**3.9 (3.3-4.3)**	**1.5 (1.1-1.8)**	**2.7 (2.2-3.3)**	**0.3 (0.2-0.4)**
**Low SDI states**	**7.9 (7.0-8.7)**	**3.5 (2.9-3.9)**	**1.6 (1.3-2.0)**	**2.5 (2.1-3.2)**	**0.3 (0.2-0.3)**	**9.6 (8.3-10.5)**	**4.3 (3.6-4.9)**	**1.8 (1.5-2.3)**	**3.0 (2.5-3.9)**	**0.4 (0.3-0.5)**
Bihar	7.1 (6.1-8.3)	3.8 (2.8-4.5)	1.2 (0.9-1.6)	2.0 (1.6-2.5)	0.1 (0.1-0.2)	9.4 (7.8-10.9)	5.1 (3.7-6.1)	1.5 (1.1-2.0)	2.6 (2.1-3.4)	0.2 (0.1-0.2)
Madhya Pradesh	7.4 (6.2-8.6)	3.2 (2.6-3.8)	1.5 (1.1-1.9)	2.2 (1.7-2.8)	0.5 (0.2-0.6)	9.0 (7.5-10.4)	4.0 (3.2-4.8)	1.7 (1.3-2.2)	2.6 (2.1-3.4)	0.6 (0.3-0.8)
Jharkhand	11.0 (9.1-12.6)	5.9 (4.9-7.1)	0.6 (0.3-1.9)	4.3 (3.1-5.2)	0.1 (0.1-0.2)	13.6 (11.0-15.7)	7.4 (6.0-8.9)	0.7 (0.4-2.2)	5.3 (3.8-6.4)	0.1 (0.1-0.3)
Uttar Pradesh	8.6 (7.3-9.9)	3.8 (3.0-4.6)	2.0 (1.4-2.5)	2.3 (1.8-3.6)	0.4 (0.2-0.5)	10.4 (8.7-12.0)	4.7 (3.7-5.7)	2.3 (1.6-2.9)	2.8 (2.1-4.4)	0.5 (0.3-0.6)
Rajasthan	7.6 (6.2-9.0)	3.0 (2.4-3.9)	2.2 (1.3-2.8)	2.1 (1.6-3.1)	0.3 (0.2-0.4)	9.3 (7.5-11.0)	3.8 (2.9-4.9)	2.5 (1.5-3.2)	2.6 (2.0-3.8)	0.3 (0.2-0.5)
Chhattisgarh	9.6 (7.2-11.2)	2.4 (1.8-3.2)	2.2 (1.6-2.9)	4.6 (3.0-5.7)	0.3 (0.2-0.5)	10.8 (8.1-12.6)	2.8 (2.1-3.7)	2.4 (1.7-3.3)	5.2 (3.4-6.4)	0.4 (0.3-0.6)
Odisha	6.8 (5.9-8.0)	2.2 (1.8-2.7)	0.9 (0.7-1.6)	3.1 (2.5-3.7)	0.5 (0.2-0.7)	7.3 (6.3-8.7)	2.4 (1.9-3.0)	0.9 (0.7-1.6)	3.3 (2.7-4.0)	0.6 (0.3-0.8)
Assam	5.0 (4.3-6.0)	1.9 (1.6-2.2)	0.7 (0.5-1.1)	2.0 (1.6-2.7)	0.4 (0.2-0.5)	6.3 (5.4-7.4)	2.5 (2.1-2.9)	0.8 (0.6-1.3)	2.5 (2.0-3.5)	0.5 (0.3-0.6)
**Middle SDI states**	**7.0 (5.9-7.7)**	**3.3 (2.7-3.7)**	**1.0 (0.8-1.3)**	**2.4 (1.9-2.8)**	**0.2 (0.2-0.2)**	**7.5 (6.2-8.2)**	**3.6 (3.0-4.1)**	**1.1 (0.8-1.3)**	**2.6 (2.0-2.9)**	**0.2 (0.2-0.3)**
Andhra Pradesh	7.5 (5.6-9.7)	4.3 (3.2-5.6)	0.8 (0.5-1.3)	2.2 (1.6-2.9)	0.1 (0.1-0.2)	7.6 (5.8-9.8)	4.5 (3.3-5.7)	0.7 (0.5-1.3)	2.2 (1.6-2.9)	0.1 (0.1-0.2)
West Bengal	5.5 (4.6-6.5)	2.9 (2.2-3.4)	0.7 (0.5-1.0)	1.6 (1.3-2.0)	0.3 (0.2-0.4)	6.1 (5.1-7.1)	3.2 (2.5-3.8)	0.7 (0.5-1.0)	1.8 (1.4-2.1)	0.4 (0.2-0.4)
Tripura	5.2 (4.2-6.4)	2.5 (1.9-3.2)	0.4 (0.2-1.0)	1.9 (1.5-2.5)	0.3 (0.2-0.4)	5.7 (4.6-7.0)	2.8 (2.2-3.5)	0.4 (0.2-1.0)	2.1 (1.6-2.6)	0.3 (0.2-0.4)
Arunachal Pradesh	4.1 (3.2-5.1)	0.3 (0.2-0.5)	0.8 (0.6-1.4)	2.6 (1.9-3.3)	0.3 (0.2-0.5)	6.0 (4.8-7.6)	0.4 (0.3-0.7)	1.2 (0.8-2.0)	3.9 (2.9-4.8)	0.5 (0.3-0.8)
Meghalaya	4.1 (3.3-5.1)	1.6 (1.3-2.0)	0.2 (0.1-0.9)	2.2 (1.5-2.8)	0.2 (0.1-0.2)	5.8 (4.7-7.1)	2.3 (1.8-2.9)	0.2 (0.1-1.2)	3.0 (2.0-3.8)	0.2 (0.2-0.3)
Karnataka	7.0 (4.8-8.0)	2.7 (2.1-3.5)	1.3 (0.8-1.7)	2.9 (1.7-3.6)	0.1 (0.0-0.2)	7.2 (5.0-8.3)	2.9 (2.2-3.7)	1.2 (0.7-1.7)	3.0 (1.8-3.7)	0.1 (0.1-0.3)
Telangana	6.6 (5.0-8.4)	3.9 (2.9-4.9)	0.7 (0.5-1.2)	1.9 (1.4-2.5)	0.1 (0.1-0.2)	7.0 (5.3-8.7)	4.1 (3.1-5.2)	0.7 (0.5-1.2)	2.0 (1.5-2.6)	0.1 (0.1-0.2)
Gujarat	7.7 (6.5-8.8)	2.3 (1.9-3.1)	1.7 (1.2-2.2)	3.4 (2.6-4.1)	0.2 (0.1-0.3)	8.1 (6.9-9.2)	2.5 (2.0-3.3)	1.7 (1.2-2.2)	3.6 (2.7-4.3)	0.2 (0.1-0.3)
Manipur	13.4 (10.0-17.2)	7.5 (5.7-9.6)	2.3 (0.8-3.3)	3.2 (1.9-4.5)	0.2 (0.1-0.3)	16.0 (12.1-20.1)	9.3 (7.1-11.8)	2.5 (0.8-3.6)	3.7 (2.1-5.1)	0.3 (0.2-0.4)
Jammu and Kashmir*	7.8 (6.2-9.1)	4.4 (3.6-5.2)	0.8 (0.6-1.2)	2.3 (1.7-2.8)	0.1 (0.1-0.1)	8.9 (7.0-10.4)	5.1 (4.1-6.0)	0.9 (0.7-1.3)	2.6 (2.0-3.2)	0.1 (0.1-0.2)
Haryana	10.3 (7.7-11.7)	5.2 (3.9-6.3)	1.4 (0.9-1.9)	3.1 (2.2-3.8)	0.5 (0.3-0.7)	11.4 (8.5-13.1)	5.9 (4.4-7.2)	1.4 (0.9-1.9)	3.4 (2.5-4.2)	0.6 (0.3-0.8)
**High SDI states**	**7.6 (6.0-8.4)**	**3.4 (2.8-4.0)**	**1.5 (0.8-2.0)**	**2.4 (1.8-2.9)**	**0.2 (0.2-0.2)**	**7.5 (6.0-8.3)**	**3.4 (2.8-4.0)**	**1.4 (0.7-1.8)**	**2.4 (1.8-2.9)**	**0.2 (0.2-0.3)**
Uttarakhand	9.5 (7.9-10.9)	4.0 (3.2-5.4)	1.8 (1.0-2.3)	3.3 (2.2-3.9)	0.5 (0.3-0.6)	10.6 (8.7-12.1)	4.5 (3.6-6.1)	1.9 (1.1-2.4)	3.6 (2.4-4.3)	0.5 (0.3-0.8)
Tamil Nadu	8.4 (6.8-9.8)	4.3 (3.4-5.1)	1.4 (0.8-1.8)	2.5 (1.9-3.1)	0.2 (0.2-0.3)	8.4 (6.8-9.7)	4.3 (3.5-5.1)	1.2 (0.7-1.6)	2.5 (1.9-3.0)	0.2 (0.2-0.3)
Mizoram	5.2 (3.9-6.6)	0.8 (0.6-1.0)	3.0 (0.8-4.3)	1.2 (0.7-3.6)	0.1 (0.1-0.2)	6.2 (4.8-8.0)	1.0 (0.7-1.3)	3.6 (0.9-5.1)	1.4 (0.9-4.3)	0.1 (0.1-0.3)
Maharashtra	6.7 (5.1-7.6)	2.9 (2.3-3.7)	1.3 (0.8-1.7)	2.2 (1.4-2.8)	0.3 (0.2-0.3)	6.8 (5.2-7.8)	3.0 (2.4-3.8)	1.2 (0.8-1.7)	2.2 (1.5-2.9)	0.3 (0.2-0.3)
Punjab	10.6 (7.8-12.2)	3.8 (2.9-4.8)	3.6 (1.0-4.6)	2.8 (1.9-4.4)	0.2 (0.2-0.3)	10.4 (7.8-11.9)	3.8 (3.0-4.7)	3.4 (0.9-4.4)	2.8 (1.9-4.3)	0.2 (0.2-0.3)
Sikkim	4.4 (3.5-5.5)	2.1 (1.6-2.6)	0.1 (0.1-0.8)	2.1 (1.6-2.7)	0.1 (0.1-0.1)	5.1 (4.1-6.4)	2.5 (2.0-3.2)	0.1 (0.1-0.8)	2.3 (1.8-3.0)	0.1 (0.1-0.2)
Nagaland	8.2 (5.6-10.7)	1.5 (1.0-3.5)	0.7 (0.5-1.7)	5.6 (2.7-7.8)	0.2 (0.2-0.5)	10.9 (7.7-14.2)	2.1 (1.3-4.8)	0.9 (0.6-2.2)	7.5 (3.5-0.4)	0.3 (0.2-0.6)
Himachal Pradesh	6.2 (5.0-7.4)	1.5 (1.1-2.4)	0.7 (0.5-1.2)	3.6 (2.4-4.5)	0.4 (0.2-0.7)	5.9 (4.9-7.1)	1.4 (1.1-2.3)	0.6 (0.4-1.1)	3.5 (2.3-4.3)	0.4 (0.2-0.7)
Union territories other than Delhi	7.7 (5.6-9.7)	4.2 (3.1-5.5)	2.0 (0.5-2.9)	1.3 (0.9-2.1)	0.1 (0.1-0.1)	8.3 (6.1-10.4)	4.8 (3.6-6.1)	2.0 (0.5-2.8)	1.4 (1.0-2.2)	0.1 (0.1-0.2)
Kerala	7.5 (5.6-8.9)	3.8 (2.9-4.7)	1.4 (0.4-2.0)	2.1 (1.4-2.7)	0.1 (0.1-0.2)	6.1 (4.6-7.2)	3.0 (2.3-3.7)	1.2 (0.3-1.7)	1.8 (1.2-2.2)	0.1 (0.1-0.2)
Delhi	5.0 (4.0-6.0)	2.0 (1.5-2.7)	0.6 (0.4-0.9)	2.2 (1.5-2.7)	0.2 (0.1-0.2)	5.7 (4.6-6.9)	2.4 (1.8-3.1)	0.6 (0.4-0.9)	2.5 (1.7-3.1)	0.2 (0.1-0.3)
Goa	6.0 (4.4-7.7)	2.9 (2.2-3.7)	1.5 (0.4-2.1)	1.5 (1.1-2.2)	0.1 (0.1-0.2)	5.2 (3.8-6.7)	2.5 (1.9-3.2)	1.2 (0.3-1.8)	1.3 (1.0-1.9)	0.1 (0.1-0.2)

SDI=Socio-demographic Index.

*The states are listed in increasing order of SDI in 2017.

†The state of Jammu and Kashmir was divided into two union territories in August 2019; as we are reporting findings up to 2017, we report findings for the state of Jammu and Kashmir.

5. Age-specific road injury death rate among males and females in India, 1990 and 2017

Age groups (years)	Road injury death rate per 100,000 among males (95% uncertainty interval)		Road injury death rate per 100,000 among females (95% uncertainty interval)	
	**1990**	**2017**	**1990**	**2017**
Under 5	9.5 (7.5-11.9)	3.0 (2.4-4.3)	7.3 (5.7-9.0)	3.5 (2.9-4.7)
5 to 9	8.1 (6.2-10.0)	2.6 (1.9-3.8)	6.1 (4.8-7.6)	2.7 (2.1-3.5)
10 to 14	5.9 (4.7-7.2)	3.2 (2.6-3.9)	3.0 (2.5-3.7)	1.6 (1.3-1.9)
15 to 19	19.7 (17.0-22.7)	11.4 (9.7-14.7)	5.3 (4.5-6.1)	2.7 (2.2-3.4)
20 to 24	31.6 (28.1-35.0)	26.5 (24.2-28.9)	6.5 (5.6-7.3)	4.0 (3.4-4.8)
25 to 29	29.9 (26.9-33.2)	27.2 (24.9-29.8)	6.6 (5.8-7.4)	4.4 (3.9-5.0)
30 to 34	29.1 (26.0-32.0)	32.1 (28.6-35.1)	7.1 (6.3-7.8)	5.3 (4.7-5.9)
35 to 39	33.5 (30.3-37.1)	36.2 (31.7-39.9)	8.1 (7.2-9.1)	6.9 (6.0-7.8)
40 to 44	32.6 (29.6-36.2)	34.7 (30.1-38.4)	8.3 (7.4-9.3)	8.7 (6.5-9.7)
45 to 49	36.6 (33.3-40.4)	38.1 (32.1-42.2)	8.7 (7.8-9.6)	9.0 (6.9-10.0)
50 to 54	39.3 (35.9-43.1)	37.2 (32.7-40.9)	12.4 (11.1-13.8)	16.1 (9.6-18.2)
55 to 59	39.7 (36.0-43.4)	43.8 (32.3-49.7)	14.6 (13.1-16.1)	16.1 (11.8-17.9)
60 to 64	44.5 (40.3-48.5)	42.0 (36.5-46.3)	20.5 (18.3-23.0)	21.9 (17.6-24.3)
65 to 69	49.6 (44.1-54.6)	46.9 (41.9-51.5)	21.2 (18.8-24.2)	21.6 (17.6-24.1)
70 to 74	53.3 (46.0-59.0)	51.6 (45.0-56.8)	25.8 (22.4-29.0)	28.1 (21.0-31.6)
75 to 79	65.8 (57.2-72.9)	65.0 (57.8-71.5)	29.3 (25.4-33.3)	27.8 (23.6-31.1)
≥80	78.4 (64.3-88.1)	94.7 (79.9-104.4)	37.3 (30.9-43.0)	48.4 (37.5-54.7)

6. Proportion of total road injury deaths in each age group among males and females in India, 1990 and 2017

Age groups (years)	Proportion of total road injury male deaths		Proportion of total road injury female deaths	
	**1990**	**2017**	**1990**	**2017**
Under 5	5.7	1.2	12.8	4.2
5 to 9	4.5	1.1	10.0	3.4
10 to 14	2.9	1.3	4.3	2.0
15 to 19	8.6	4.8	6.7	3.4
20 to 24	12.4	10.3	7.5	4.8
25 to 29	10.1	9.7	6.8	4.9
30 to 34	8.6	10.8	6.3	5.6
35 to 39	8.9	10.6	6.4	6.4
40 to 44	7.3	9.0	5.4	7.0
45 to 49	6.9	8.6	4.6	6.3
50 to 54	6.0	6.8	5.3	9.4
55 to 59	5.1	6.5	5.4	7.9
60 to 64	4.6	5.3	6.0	9.4
65 to 69	3.4	4.7	4.3	7.4
70 to 74	2.2	3.5	3.5	6.6
75 to 79	1.5	2.7	2.4	4.6
≥80	1.3	3.0	2.2	6.7
Total	100	100	100	100

7. Road injury deaths and their proportion by type of road users among males and females in the states of India, 2017

States of India*	Total number of road injury deaths (95% uncertainty intervals)		Percentage of total road injury deaths in each sex									
			Pedestrian		Motor cyclist		Motor vehicle occupant		Cyclist		Other RTI	
	Males	Females	Males	Females	Males	Females	Males	Females	Males	Females	Males	Females
**India**	**167,830 (154,532-178,409)**	**51,046 (43,939-54,572)**	**32.1**	**44.8**	**34.6**	**18.6**	**24.7**	**32.1**	**8.0**	**3.6**	**0.6**	**0.8**
Bihar	9,851 (8,463-11,469)	4,178 (3,557-4,841)	47.3	53.3	24.5	17.0	21.5	27.6	6.3	1.5	0.4	0.6
Madhya Pradesh	10,705 (9,322-12,322)	3,073 (2,583-3,558)	26.6	43.1	34.3	20.2	30.5	29.2	7.9	6.4	0.7	1.2
Jharkhand	4,434 (3,873-5,238)	2,045 (1,694-2,350)	36.0	53.8	23.3	5.3	28.2	39.5	12.2	1.0	0.3	0.4
Uttar Pradesh	32,746 (28,420-37,453)	9,627 (8,160-11,063)	33.3	44.2	35.1	23.0	21.5	27.1	9.7	4.7	0.4	1.1
Rajasthan	10,709 (9,022-12,434)	2,970 (2,418-3,488)	25.6	39.9	41.1	28.6	26.5	27.5	5.9	3.5	1.0	0.5
Chhattisgarh	4,299 3,407-5,024)	1,445 (1,091-1,689)	25.5	25.1	41.7	22.6	20.9	47.9	10.5	3.6	1.4	0.8
Odisha	5,193 (4,583-5,830)	1,601 (1,386-1,899)	20.3	31.8	36.2	13.6	28.0	45.9	14.1	7.6	1.3	1.0
Assam	3,380 (2,985-3,836)	877 (755-1056)	31.0	38.2	28.8	13.7	28.7	39.7	11.1	7.7	0.4	0.7
Andhra Pradesh	6,585 (5,115-8,336)	2,038 (1,535-2,635)	37.4	58.1	32.4	10.3	24.3	28.9	4.7	1.5	1.1	1.2
West Bengal	9,584 (8,189-11,088)	2,737 (2,287-3,202)	47.1	51.6	17.9	12.6	24.0	29.2	10.3	5.7	0.6	0.9
Tripura	417 (327-543)	104 (84-129)	35.2	49.0	15.7	6.8	37.6	37.2	11.1	5.9	0.4	1.0
Arunachal Pradesh	146 (113-196)	34 (27-42)	22.1	6.7	29.3	20.8	41.9	64.1	6.3	7.2	0.3	1.2
Meghalaya	194 (155-251)	69 (56-85)	25.9	38.1	16.2	4.6	50.7	52.5	6.6	3.8	0.6	0.9
Karnataka	9,165 (7,202-10,657)	2,345 (1,595-2,695)	27.4	38.8	40.9	17.9	26.6	41.2	4.6	1.3	0.5	0.7
Telangana	4,256 (3,356-5,399)	1,311 (989-1,658)	36.3	58.4	33.5	10.3	24.6	28.7	4.7	1.5	0.9	1.1
Gujarat	6,759 (5,867-7,706)	2,570 (2,177-2,938)	21.7	30.4	40.4	22.3	31.8	44.6	5.4	2.2	0.7	0.5
Manipur	399 (317-505)	228 (170-292)	39.3	55.9	25.9	17.3	29.1	24.1	5.0	1.5	0.5	1.1
Jammu and Kashmir†	2,274 (1,926-2,648)	511 (408-598)	36.1	56.6	25.3	10.8	34.6	29.9	3.6	1.1	0.4	1.5
Haryana	4,514 (3,918-5,134)	1,406 (1,051-1,604)	21.3	50.9	44.6	13.4	24.4	30.2	8.9	5.1	0.7	0.4
Uttarakhand	2,270 (1,875-2,669)	528 (437-605)	28.2	41.7	35.8	18.7	27.0	34.3	8.7	4.8	0.3	0.4
Tamil Nadu	13,365 (11,734-15,061)	3,408 (2,754-3,946)	32.6	50.7	35.5	16.1	21.9	29.6	9.3	2.5	0.6	1.1
Mizoram	141 (112-176)	32 (24-41)	14.4	14.9	43.7	58.1	36.2	23.5	4.9	2.1	0.9	1.4
Maharashtra	12,925 (11,296-14,694)	4,019 (3,054-4,594)	29.8	43.8	42.5	19.2	22.8	32.6	4.1	3.9	0.8	0.5
Punjab	5,803 (5,003-6,626)	1,549 (1,147-1,788)	27.8	35.9	36.6	33.8	23.4	26.9	11.9	2.3	0.3	1.2
Sikkim	59 (47-73)	14 (11-18)	27.2	46.9	18.6	3.1	48.9	47.5	4.4	2.0	0.9	0.5
Nagaland	141 (109-179)	79 (54-103)	24.3	18.8	35.0	9.1	36.6	68.7	3.4	2.9	0.8	0.5
Himachal Pradesh	1,149 (866-1,360)	230 (187-275)	21.5	23.7	29.7	10.6	42.4	58.6	6.0	6.7	0.4	0.4
Union territories other than Delhi	361 (281-450)	135 (99-172)	32.5	55.4	37.7	26.1	21.6	17.0	7.7	1.2	0.5	0.4
Kerala	4,243 (3,441-4,961)	1,394 (1,032-1,652)	41.3	50.1	34.4	19.0	16.3	28.3	7.7	1.9	0.3	0.7
Delhi	1,621 (1,307-1,935)	446 (356-534)	32.7	40.0	30.2	12.4	26.8	43.2	9.5	3.4	0.7	1.1
Goa	143 (109-178)	46 (34-59)	36.2	48.5	40.8	24.1	18.4	24.8	4.1	2.1	0.4	0.5

RTI=Road traffic injury. SDI=Socio-demographic Index.
*The states are listed in increasing order of SDI in 2017.
†The state of Jammu and Kashmir was divided into two union territories in August 2019; as we are reporting findings up to 2017, we report findings for the state of Jammu and Kashmir.

8. Age-specific road injury death rate by type of road users in India, 2017

Both sexes combined

Age groups (years)	Road injury death rate per 100,000 population (95% uncertainty interval)				
	Overall	Pedestrian	Motorcyclist	Motor vehicle occupant	Cyclist
Under 5	3.2 (2.7-4.4)	1.7 (1.4-2.3)	0.4 (0.3-0.6)	1.0 (0.8-1.4)	0.1 (0.0-0.1)
5 to 9	2.6 (2.1-3.5)	1.7 (1.3-2.1)	0.2 (0.1-0.3)	0.6 (0.4-0.8)	0.2 (0.1-0.2)
10 to 14	2.4 (2.0-2.9)	1.2 (1.0-1.5)	0.3 (0.2-0.4)	0.6 (0.5-0.8)	0.3 (0.2-0.3)
15 to 19	7.2 (6.3-9.2)	1.7 (1.4-2.0)	2.8 (2.2-3.9)	2.0 (1.6-2.6)	0.6 (0.4-0.6)
20 to 24	15.6 (14.3-16.9)	3.4 (3.0-3.9)	7.1 (5.8-8.1)	4.4 (3.8-5.6)	0.7 (0.4-0.7)
25 to 29	16.1 (14.8-17.5)	3.7 (3.2-4.2)	6.9 (5.5-7.9)	4.7 (4.1-6.0)	0.8 (0.5-0.8)
30 to 34	19.1 (17.3-20.7)	4.5 (3.9-5.4)	8.3 (6.6-9.4)	5.1 (4.4-6.7)	1.1 (0.8-1.1)
35 to 39	21.8 (19.4-23.8)	6.4 (5.4-7.5)	8.1 (6.1-9.4)	6.1 (5.2-7.8)	1.1 (0.7-1.1)
40 to 44	22.0 (19.1-24.1)	6.6 (5.4-7.7)	7.9 (5.8-9.1)	5.7 (4.6-7.4)	1.8 (1.2-1.8)
45 to 49	23.9 (20.3-26.3)	7.1 (5.8-8.6)	7.9 (5.8-9.3)	6.6 (5.2-8.5)	2.0 (1.4-2.0)
50 to 54	26.8 (22.0-29.2)	9.3 (7.3-10.9)	7.7 (5.7-8.9)	7.2 (5.4-9.1)	2.4 (1.7-2.4)
55 to 59	30.0 (22.8-33.3)	11.9 (8.7-14.3)	7.5 (4.9-9.3)	8.4 (5.9-10.9)	1.8 (1.2-1.8)
60 to 64	31.8 (27.3-34.3)	15.1 (12.2-16.9)	5.9 (4.4-6.9)	8.1 (6.6-9.9)	2.6 (1.8-2.6)
65 to 69	34.0 (30.5-36.7)	17.1 (14.8-19.1)	5.9 (4.3-6.8)	7.4 (6.2-9.4)	3.2 (2.3-3.2)
70 to 74	39.5 (33.7-43.0)	21.3 (17.6-23.8)	5.5 (4.0-6.5)	8.6 (6.7-11.2)	3.7 (2.7-3.7)
75 to 79	44.7 (40.8-48.1)	25.3 (22.1-27.8)	3.9 (2.8-4.5)	10.2 (8.9-12.7)	4.6 (3.5-4.6)
≥80	68.3 (57.3-73.9)	39.4 (31.7-44.4)	8.6 (6.2-10.0)	15.0 (11.8-18.4)	4.0 (3.0-4.0)

Males

Age groups (years)	Road injury death rate per 100,000 population (95% uncertainty interval)				
	Overall	Pedestrian	Motorcyclist	Motor vehicle occupant	Cyclist
Under 5	3.0 (2.4-4.3)	1.6 (1.2-2.2)	0.3 (0.2-0.5)	1.0 (0.7-1.4)	0.1 (0.0-0.1)
5 to 9	2.6 (1.9-3.8)	1.3 (1.0-1.8)	0.3 (0.2-0.4)	0.6 (0.4-1.0)	0.3 (0.2-0.3)
10 to 14	3.2 (2.6-3.9)	1.6 (1.3-2.0)	0.4 (0.3-0.5)	0.7 (0.6-1.0)	0.4 (0.3-0.4)
15 to 19	11.4 (9.7-14.7)	2.5 (1.9-3.1)	4.9 (3.8-7.1)	2.9 (2.2-3.9)	1.1 (0.6-1.1)
20 to 24	26.5 (24.2-28.9)	5.4 (4.5-6.4)	12.8 (10.4-14.6)	7.1 (6.1-9.5)	1.1 (0.7-1.1)
25 to 29	27.2 (24.9-29.8)	6.0 (5.1-7.1)	12.4 (9.6-14.2)	7.3 (6.2-10.0)	1.4 (0.9-1.4)
30 to 34	32.1 (28.6-35.1)	7.4 (6.0-8.9)	14.4 (11.1-16.6)	8.5 (7.1-11.7)	1.9 (1.3-1.9)
35 to 39	36.2 (31.7-39.9)	9.9 (7.9-12.0)	14.5 (10.7-17.0)	9.6 (8.0-12.9)	2.1 (1.4-2.1)
40 to 44	34.7 (30.1-38.4)	9.7 (8.0-11.7)	13.4 (9.9-15.7)	8.1 (6.7-11.4)	3.4 (2.2-3.4)
45 to 49	38.1 (32.1-42.2)	11.2 (9.1-13.8)	13.5 (9.7-16.2)	9.5 (7.4-13.1)	3.6 (2.3-3.6)
50 to 54	37.2 (32.7-40.9)	12.5 (10.0-14.5)	11.3 (8.3-13.4)	8.7 (7.3-11.7)	4.4 (2.9-4.4)
55 to 59	43.8 (32.3-49.7)	16.0 (11.2-20.0)	12.3 (7.6-15.8)	11.8 (7.9-16.3)	3.3 (2.1-3.3)
60 to 64	42.0 (36.5-46.3)	18.9 (15.2-21.8)	8.3 (5.8-10.0)	9.8 (7.8-12.9)	4.9 (3.3-4.9)
65 to 69	46.9 (41.9-51.5)	23.1 (19.5-26.3)	8.7 (6.0-10.6)	9.0 (7.4-12.3)	5.5 (3.6-5.5)
70 to 74	51.6 (45.0-56.8)	26.1 (21.6-29.8)	7.9 (5.3-9.7)	10.7 (8.5-14.5)	6.0 (3.9-6.0)
75 to 79	65.0 (57.8-71.5)	40.3 (34.2-45.1)	5.1 (3.4-6.2)	11.7 (9.6-15.9)	7.0 (4.6-7.0)
≥80	94.7 (79.9-104.4)	53.9 (43.4-61.4)	16.0 (11.2-18.9)	15.8 (12.0-20.7)	6.9 (4.7-6.9)

Females

Age groups (years)	Road injury death rate per 100,000 population (95% uncertainty interval)				
	Overall	Pedestrian	Motorcyclist	Motor vehicle occupant	Cyclist
Under 5	3.5 (2.9-4.7)	1.8 (1.4-2.5)	0.6 (0.4-0.8)	1.0 (0.8-1.4)	0.03 (0.02-0.06)
5 to 9	2.7 (2.1-3.5)	2.0 (1.5-2.5)	0.1 (0.1-0.2)	0.5 (0.4-0.7)	0.1 (0.1-0.1)
10 to 14	1.6 (1.3-1.9)	0.8 (0.7-1.0)	0.2 (0.1-0.2)	0.4 (0.3-0.6)	0.1 (0.1-0.2)
15 to 19	2.7 (2.2-3.4)	0.9 (0.7-1.1)	0.5 (0.3-0.7)	1.0 (0.8-1.4)	0.2 (0.2-0.3)
20 to 24	4.0 (3.4-4.8)	1.3 (1.0-1.6)	1.1 (0.7-1.4)	1.4 (1.2-1.9)	0.1 (0.1-0.2)
25 to 29	4.4 (3.9-5.0)	1.2 (0.9-1.4)	1.2 (0.9-1.5)	1.9 (1.6-2.3)	0.1 (0.1-0.2)
30 to 34	5.3 (4.7-5.9)	1.6 (1.3-1.9)	1.9 (1.5-2.3)	1.6 (1.3-1.9)	0.2 (0.2-0.3)
35 to 39	6.9 (6.0-7.8)	2.8 (2.3-3.3)	1.5 (1.1-1.9)	2.5 (2.0-3.0)	0.05 (0.03-0.06)
40 to 44	8.7 (6.5-9.7)	3.3 (2.5-4.0)	2.0 (1.4-2.7)	3.1 (2.1-3.8)	0.2 (0.2-0.2)
45 to 49	9.0 (6.9-10.0)	2.9 (2.3-3.5)	2.0 (1.4-2.6)	3.6 (2.6-4.3)	0.4 (0.4-0.5)
50 to 54	16.1 (9.6-18.2)	6.0 (4.0-7.8)	3.9 (2.1-5.4)	5.6 (2.9-7.4)	0.5 (0.5-0.6)
55 to 59	16.1 (11.8-17.9)	7.8 (5.9-9.3)	2.8 (1.8-3.6)	5.1 (3.4-6.4)	0.4 (0.4-0.5)
60 to 64	21.9 (17.6-24.3)	11.3 (9.1-13.1)	3.6 (2.5-4.6)	6.5 (4.8-7.9)	0.5 (0.5-0.6)
65 to 69	21.6 (17.6-24.1)	11.4 (9.3-13.0)	3.2 (2.2-4.0)	5.9 (4.7-7.3)	1.1 (1.1-1.4)
70 to 74	28.1 (21.0-31.6)	16.7 (12.9-19.3)	3.1 (2.0-4.2)	6.6 (4.5-8.4)	1.6 (1.6-2.0)
75 to 79	27.8 (23.6-31.1)	12.9 (10.8-14.8)	2.9 (2.1-3.6)	9.0 (7.7-11.3)	2.5 (2.5-3.2)
≥80	48.4 (37.5-54.7)	28.6 (22.1-32.8)	3.0 (2.0-3.9)	14.5 (10.8-18.1)	1.8 (1.8-2.3)

Other road injury are not shown as their rates are negligible.

9. Relationship between the age-standardised death rate for overall, motor vehicle occupant, motorcyclist, pedestrian, and cyclist road injury with the per capita GDP in the states of India, 2017

Overall road injury

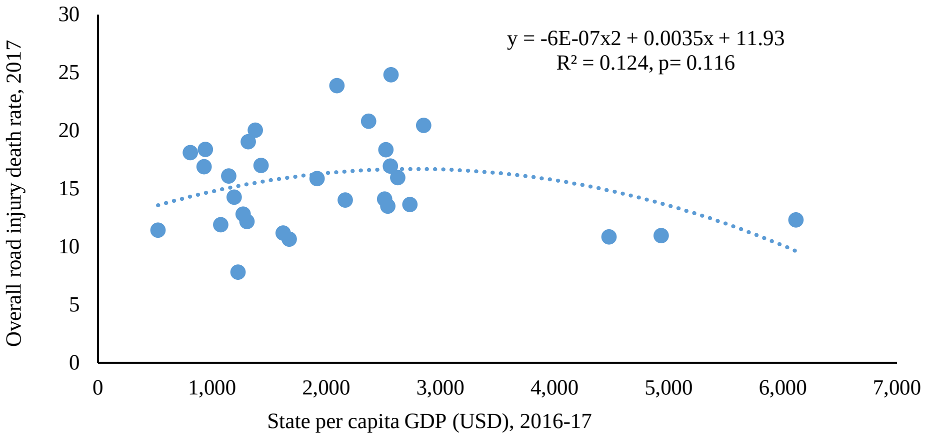

Motor vehicle occupant road injury

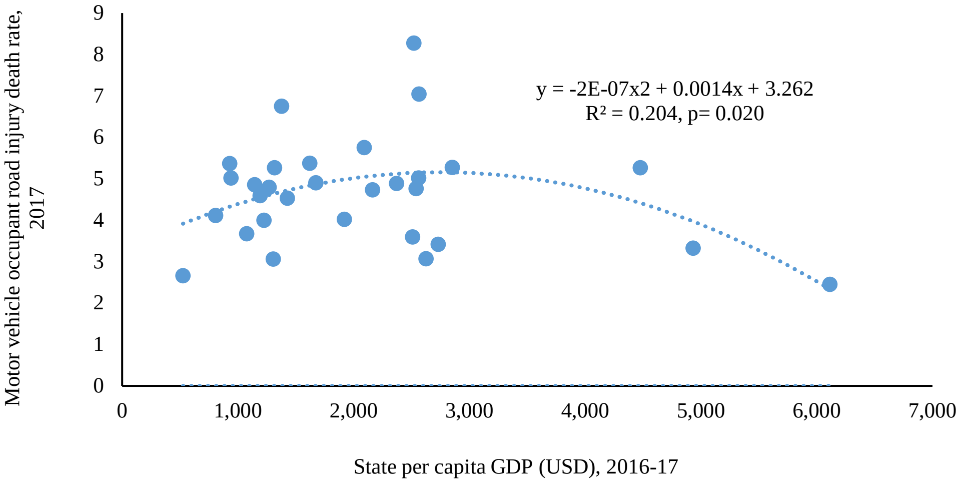

Motorcyclist road injury

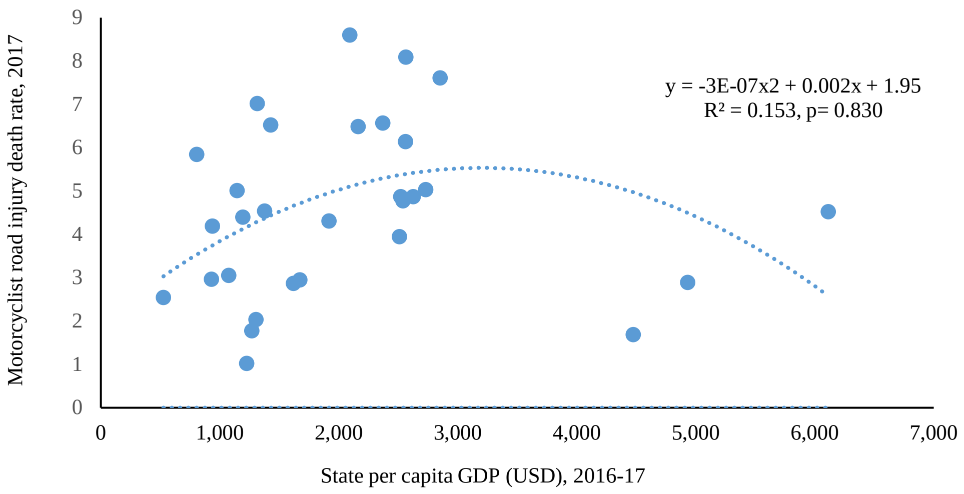

Pedestrian road injury

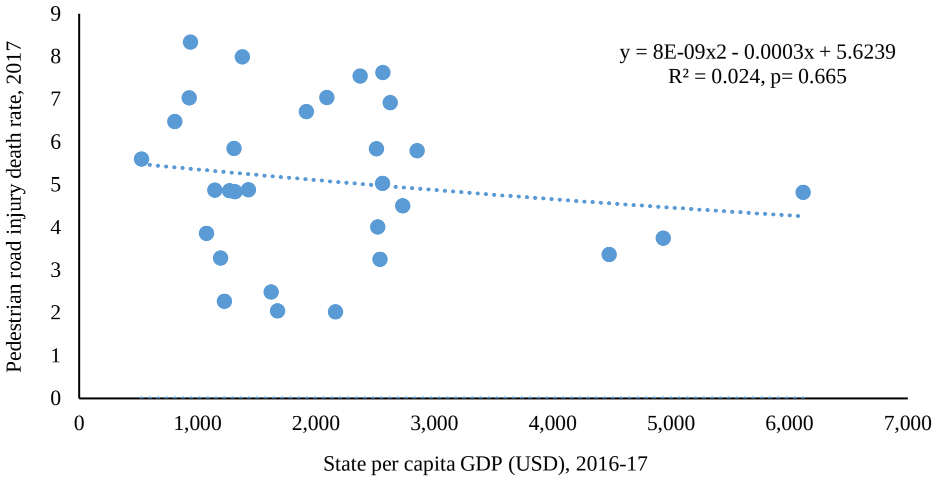

Cyclist road injury

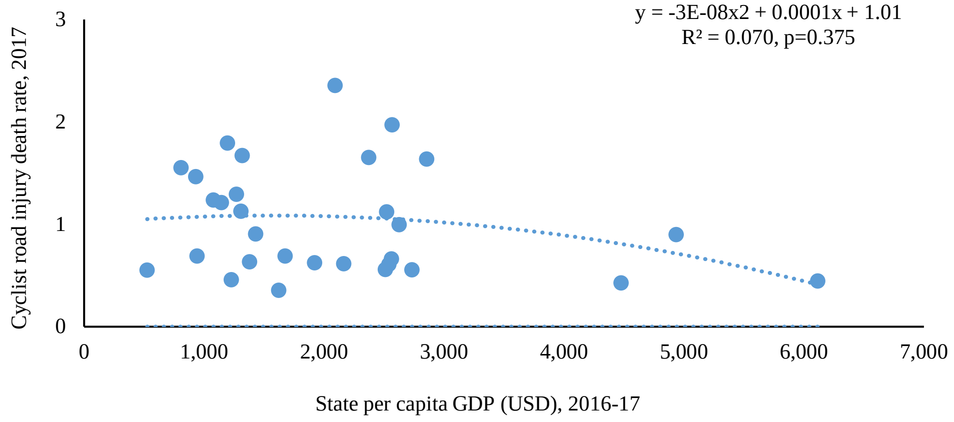

GDP=Gross domestic product.

10. Relationship between the age-standardised death rate for overall, motor vehicle occupant, motorcyclist, pedestrian and cyclist road injury with the per capita vehicles in the states of India, 2017

Overall road injury

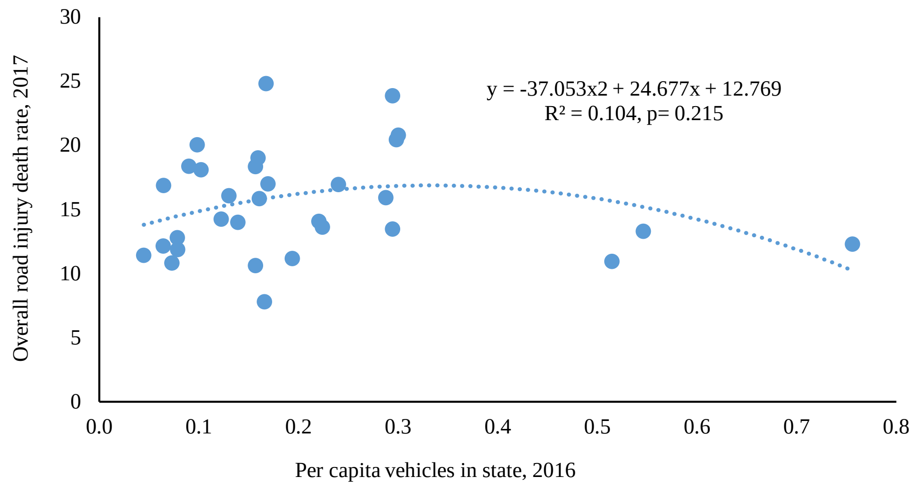

Motor vehicle occupant road injury

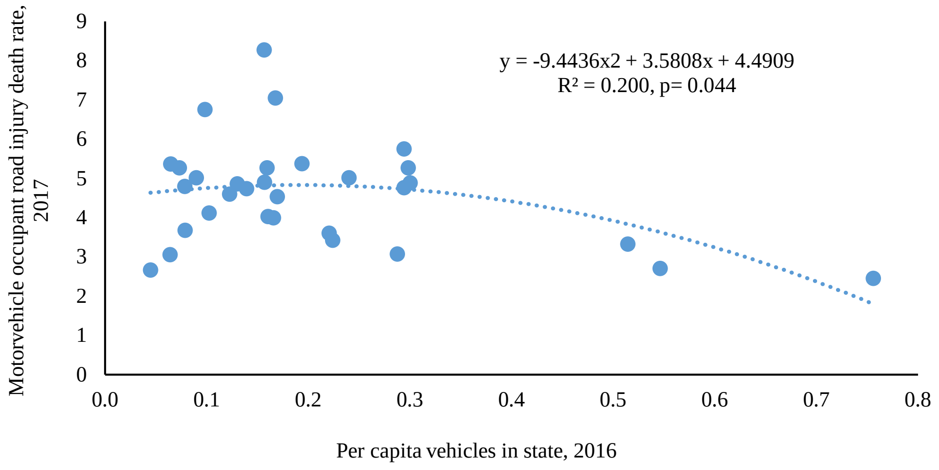

Motorcyclist road injury

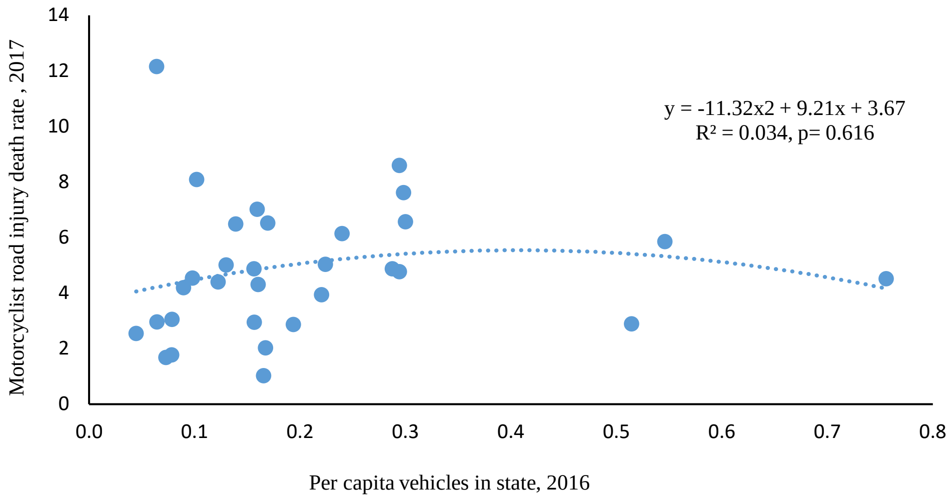

Pedestrian road injury

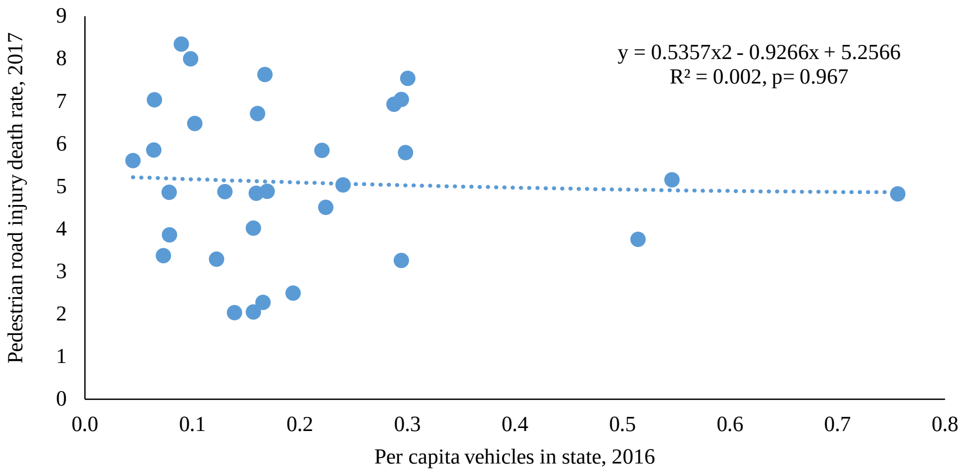

Cyclist road injury

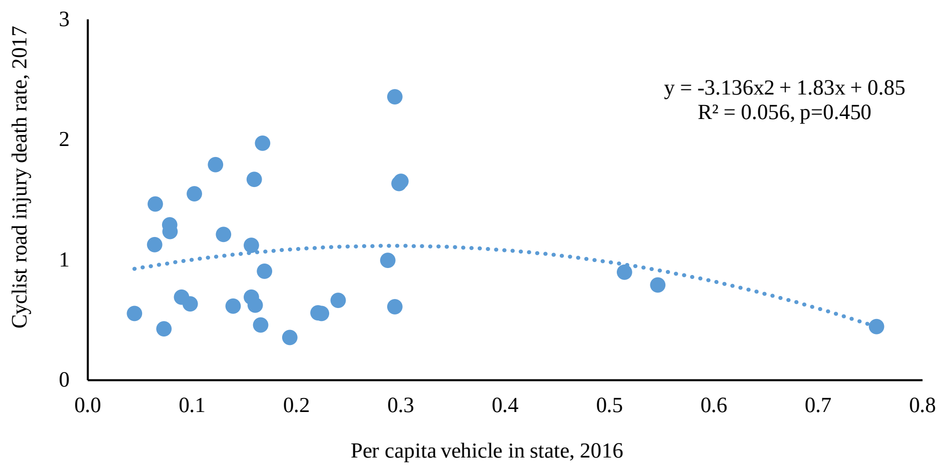

11. Gap between the projected road injury ASDR versus the SDG target for the states of India in 2020 and 2030

**States of India***	**Road injury ASDR per 100,000 in 2015**	**SDG 2020 target for road injury ASDR per 100,000**[†]	**Road injury ASDR per 100,000 in 2020, if trends up to 2017 continue**	**Road injury ASDR per 100,000 in 2030, if trends up to 2017 continue**	**Gap between projected road injury ASDR per 100,000 in 2020 versus the SDG 2020 target**	**Gap between projected road injury ASDR per 100,000 in 2030 versus the SDG 2020 target**
India	17.8	8.9	16.8	15.6	7.9	6.7
Bihar	15.2	7.6	14.1	13.1	6.5	5.5
Madhya Pradesh	19.3	9.6	18.5	18.8	8.9	9.2
Jharkhand	21.7	10.9	19.0	16.3	8.1	5.4
Uttar Pradesh	22.0	11.0	21.2	19.9	10.2	8.9
Rajasthan	20.3	10.1	19.7	19.3	9.6	9.2
Chhattisgarh	21.7	10.8	21.6	23.5	10.8	12.7
Odisha	15.9	8.0	14.6	14	6.6	6.0
Assam	14.2	7.1	13.1	11.6	6.0	4.5
Andhra Pradesh	16.4	8.2	15.5	14.5	7.3	6.3
West Bengal	13.1	6.5	12.0	10.4	5.5	3.9
Tripura	13.7	6.9	13.0	12	6.1	5.1
Arunachal Pradesh	14.8	7.4	13.6	11.6	6.2	4.2
Meghalaya	10.3	5.2	9.8	9.2	4.6	4.0
Karnataka	16.7	8.4	17.2	18.4	8.8	10.0
Telangana	15.2	7.6	13.7	11.7	6.1	4.1
Gujarat	14.7	7.3	13.7	12.7	6.4	5.4
Manipur	21.8	10.9	21.3	21.4	10.4	10.5
Jammu and Kashmir[‡]	22.1	11.0	21.0	18.5	10.0	7.5
Haryana	23.3	11.7	21.5	20.2	9.8	8.5
Uttarakhand	27.2	13.6	25.1	21.7	11.5	8.1
Tamil Nadu	21.8	10.9	19.6	18.2	8.7	7.3
Mizoram	16.1	8.0	15.6	14.5	7.6	6.5
Maharashtra	14.3	7.1	13.1	11.5	6.0	4.4
Punjab	23.9	11.9	22.5	21	10.6	9.1
Sikkim	12.1	6.1	11.2	9.7	5.1	3.6
Nagaland	14.5	7.2	13.6	12.2	6.4	5.0
Himachal Pradesh	16.9	8.5	17.4	17.1	8.9	8.6
Union territories other than Delhi	14.4	7.2	13.9	13.5	6.7	6.3
Kerala	14.1	7.1	13.8	13.4	6.7	6.3
Delhi	12.4	6.2	11	8.8	4.8	2.6
Goa	11.4	5.7	10.9	10.4	5.2	4.7

ASDR=Age-standardised death rate.
*The states are listed in increasing order of SDI in 2017.
[†]SDG target of halve the RI ASDR from 2015 to 2020.
[‡]The state of Jammu and Kashmir was divided into two union territories in August 2019; as we are reporting findings up to 2017, we report findings for the state of Jammu and Kashmir.
